# Pseudo-adsorption and long-range redox coupling during oxygen reduction reaction on single atom electrocatalyst

**DOI:** 10.1038/s41467-022-29357-7

**Published:** 2022-04-01

**Authors:** Jie-Wei Chen, Zisheng Zhang, Hui-Min Yan, Guang-Jie Xia, Hao Cao, Yang-Gang Wang

**Affiliations:** 1 Department of Chemistry, Southern University of Science and Technology, 518055 Shenzhen, Guangdong China; 2 Guangdong Provincial Key Laboratory of Catalysis, Southern University of Science and Technology, 518055 Shenzhen, Guangdong China; 3 Department of Chemistry and Biochemistry, University of California, Los Angeles, 607 Charles E. Young Drive East, Los Angeles, CA 90095 USA

**Keywords:** Electrocatalysis, Electrocatalysis, Molecular dynamics

## Abstract

Fundamental understanding of the dynamic behaviors at the electrochemical interface is crucial for electrocatalyst design and optimization. Here, we revisit the oxygen reduction reaction mechanism on a series of transition metal (M = Fe, Co, Ni, Cu) single atom sites embedded in N-doped nanocarbon by ab initio molecular dynamics simulations with explicit solvation. We have identified the dissociative pathways and the thereby emerged solvated hydroxide species for all the proton-coupled electron transfer (PCET) steps at the electrochemical interface. Such hydroxide species can be dynamically confined in a “pseudo-adsorption” state at a few water layers away from the active site and respond to the redox event at the catalytic center in a coupled manner within timescale less than 1 ps. In the PCET steps, the proton species (in form of hydronium in neutral/acidic media or water in alkaline medium) can protonate the pseudo-adsorbed hydroxide without needing to travel to the direct catalyst surface. This, therefore, expands the reactive region beyond the direct catalyst surface, boosting the reaction kinetics via alleviating mass transfer limits. Our work implies that in catalysis the reaction species may not necessarily bind to the catalyst surface but be confined in an active region.

## Abstract (web summary)

The reaction region is commonly considered to be the direct catalyst surface. Here, the authors challenge this view and use molecular dynamics simulations to reveal a solvated hydroxide species dynamically confined in a pseudo-adsorption state at a few water layers away from the active site during oxygen reduction reaction on single atom electrocatalyst.

## Introduction

In the field of electrocatalysis, oxygen reduction reaction (ORR) has been extensively studied for the purpose of developing renewable-energy technologies, such as fuel cells and metal–air batteries^1–4^. However, due to the sluggish reaction kinetics, the ORR usually requires using highly efficient platinum-based catalysts, which are of high costs and scarce reserves. To achieve the large-scale commercialization of the fuel cell technologies, reducing the cathode Pt loading or even completely replacing it with non-precious metal catalysts are highly desirable. In recent years, the single atom catalysts (SACs) is becoming a hot spot since the Pt_1_/FeO_*x*_ was firstly reported to exhibit maximized atomic efficiency and superior catalytic activity towards CO oxidation^5,6^. The concept readily translates to the field to electrocatalysis, and a family of single transition metal-embedded N-doped nanocarbon (M−N_*x*_/C, M = Fe, Co, Ni, etc.) catalysts receives increasing research attention for their low price, easy fabrication, electric conductance, high electrochemical activity towards a wide range of electrocatalytic reactions, and long-term stability under practical working environments^6–13^.

Fundamental understanding of the electrocatalytic chemistry in realistic environment is crucial in advancing the development and optimization of the single atom catalyst. To explore the prominent catalytic performance of M–N_*x*_/C single atom catalysts in ORR, extensive efforts including both experimental and theoretical studies have been made in recent years^2,14–20^. The mechanisms are generally considered to be two-electron or four-electron transfer pathways^21^. The two-electron pathway leads to the formation of hydrogen peroxide (H_2_O_2_) as the final product with *OOH being the only reaction intermediate, while four-electron pathway results in water formation in the final step. Specially, for the four-electron pathway, both associative and dissociative mechanisms are observed depending on whether O_2_ dissociate or not prior to the electron transfer. For example, ORR occurred on metal surface majorly via dissociative mechanism thanks to a plenty of metal sites adjacent to the adsorption site, while for M–N_*x*_/C catalysts the associative mechanism is the major pathway^21–24^.

Under realistic conditions, the underlying elementary steps of ORR take place at the electrode–liquid interface where the reactants and intermediates not only interact with the catalyst substrate but also dynamically communicate with the solvent molecules. Although myriads of reports have shown the influence of solvent environment on many aspects of the catalysis such as adsorption configurations, reaction free energetics (especially through the entropy contribution) and even the reaction pathways, mechanistic studies considering solvation effects remain to be a challenging task due to the complexity of models and high computational cost. To describe the solvent environment, there are two theoretical approaches in DFT calculations: the explicit model and the implicit model. Most of the existing theoretical reports on the ORR on M–N_*x*_–C catalysts used the implicit model^25^, which expresses the effect of solvent environment by a continuous polarizable medium. However, implicit models are not able to describe hydrogen bonding and ion distributions, leading to underestimation of solvation effects. It is reported that both Poisson–Boltzmann (PB) and generalized Born (GB) model have shown considerable differences in the estimation of free energetics^26^. In contrast to the implicit model, explicit model adds explicit water molecules around the reactant or above the surface to directly describe the interaction between the reaction intermediates and the solvent molecules. Recently, Bao et al. ^27^ studied the ORR mechanism on nitrogen-doped grapheme by including 41 H_2_O molecules with a density of 1 g/cm^3^ to simulate the water environment, and showed the hydrogen bonding plays an important role in the polarization of O_2_. Liu et al. ^14^ simulated the free energetics of the ORR on Mn/CN_4_ single-atom catalyst by covering a layer of water molecules on the substrate. Although the theoretical studies with the explicit model indeed provide insight into the catalytic behavior of the ORR at the electrochemical interface, it is still grand challenge to explore the dynamic properties occurring at the solid–liquid interface by placing a few H_2_O molecules or a single aqueous layer above the metal surface other than bulk water.

Herein, we have performed ab initio molecular dynamics simulations with explicit water molecules to probe the dynamic properties of the ORR processes on a series of M-N_4_/C (M = Fe, Co, Ni, Cu) SACs in aqueous media. We have identified a non-traditional dissociative pathway where a hydroxide anion is released after the first proton coupled electron transfer step instead of staying in the *OOH form. The hydroxide species can also emerge in later PCET steps, diverge the reaction pathway from the traditional mechanism. The released hydroxide anion neither returns to the catalytic center nor diffuses away but confined in a solvated “pseudo-adsorption” state at ca. 10 Å away from the catalyst surface. In addition, the solvent water is found to dynamically coordinate to the metal center in an adaptive manner, adjusting its binding strength as the ORR proceeds and promoting an octahedral coordination field. Based on the discoveries, we propose a revised four-electron transfer mechanism which shows better agreement with the experimental overpotential of ORR. The pseudo-adsorption and dynamic water coordination could be universal phenomena in aqueous electrocatalysis using SACs, and we stress the necessity of including realistic water environments in mechanistic study.

## Results

### Solvation-stabilized dissociative pathway

On M-N_4_/C single atom catalyst, the ORR is usually considered to follow a 4*e*^−^ associative pathway. The detailed elementary steps after O_2_ adsorption are the following (the H^+^ stands for proton provided by the hydronium/water pair in acidic/neutral media or by water/hydroxide pair in alkaline medium): (i) *O_2_ + H^+^ + *e*^*−*^ → *OOH; (ii) *OOH + H^+^ + *e*^*−*^ → H_2_O(l) + *O; (iii) *O + H^+^ + *e*^*−*^ → *OH; (vi) *OH + H^+^ + *e*^*−*^ → * + H_2_O(l). Note that each proton transfer (PT) step is considered coupled with an electron transfer (ET) step in a proton coupled electron transfer (PCET) manner. During the associative pathway, the adsorbed species *O_2_, *OOH, *O, and *OH are the main reaction intermediates.

**Fig. 1:**
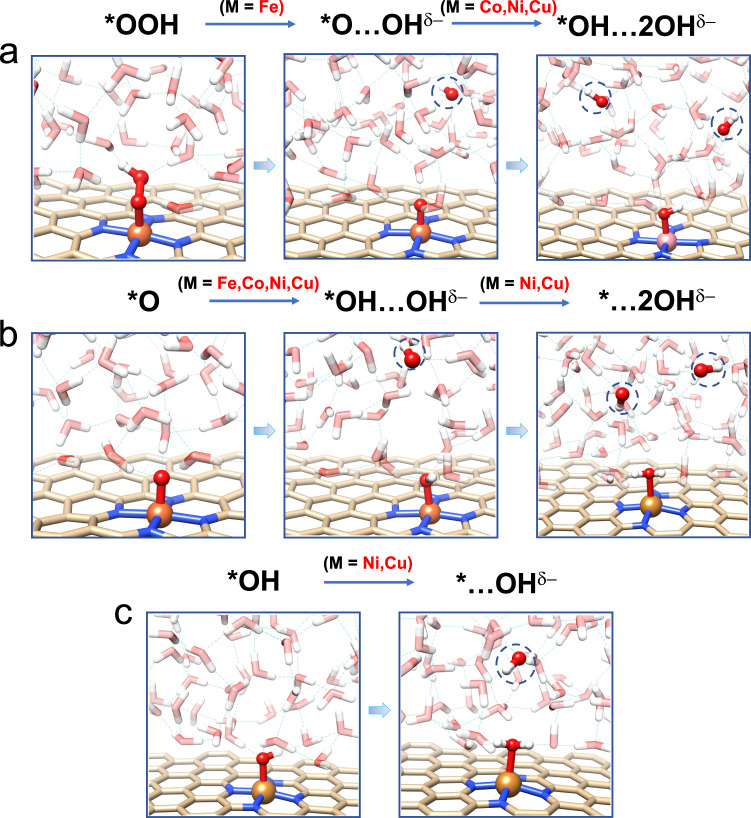
MD Snapshots of reaction intermediates transforming to the pseudo-adsorption state. **a** *OOH species forms hydrogen bonds with the surrounding water molecule and quickly dissociates into an adsorbed oxygen (*O) species and a hydroxyl dissolving into the water on Fe–N_4_/C catalyst. Further protonation could be observed on Co–N_4_/C, Ni–N_4_/C, and Cu–N_4_/C catalysts. **b** *O catches proton from solvent environment to form *OH…OH^δ−^. For Ni–N_4_/C and Cu–N_4_/C catalyst, further protonation to form *…**2**OH^δ−^ was observed. **c** the protonation of *OH could be observed on Ni–N_4_/C and Cu–N_4_/C catalyst. The Fe, Co, Cu, N, C, O, and H atoms are colored in brown, pink, orange, blue, light yellow, red, and white, respectively.

To explore the effect of solvation on the ORR chemistry, we first performed ab initio molecular dynamics (AIMD) simulations on each ORR intermediate on Fe-N_4_/C by exposing them to explicit water molecules to describe the liquid environment (see computational models in Supplementary Fig. 1). The radial distribution functions (RDFs) of O–H and O–O (Supplementary Fig. 2) are in well agreement with experimental data^28^, which validates our model in correctly describing the behavior of liquid water. The representative AIMD snapshots for each intermediate are shown in Fig. 1 and Supplementary Fig. 3. For the *O_2_ species, our simulations show that it has a side-on adsorption configuration with its outer O forming about 3 hydrogen bonds with the surrounding water molecules. The Fe–O–O species stays rather stable during the 30 ps simulation without any desorption, proving the nature of such binding as chemisorption. The averaged bond length of O–O here is 1.39 Å, which is significant longer than the bond length of O=O in molecular oxygen (~1.21 Å), indicating the formation of an [O_2_]^−^ peroxide species (Supplementary Fig. 3a). The Bader charge analysis further confirms that the adsorbed O_2_ species possess a charge of −0.96*e* and the catalyst has a charge of +1.06*e*, which implies that the adsorbed oxygen species is in fact pre-activated by the Fe-N_4_ through back-donation of Fe 3*d* electron before electrochemical process starts. As reported in previous theoretical studies, the first proton coupled electron transfer step leads to the formation of *OOH species. However, it is observed in our AIMD simulations in Fig. 1a that the *OOH species is indeed unstable under exposure to liquid water at room temperature (300 K). Solvated by the surrounding water molecules through hydrogen bonds, the O–O bond in *OOH is weakened and quickly dissociates into an adsorbed oxygen (*O) species and a OH species. The *OOH dissociation event only takes ~1 ps to complete. The Bader charge analysis further shows that the resulted OH species possesses a net charge of ca. −1.0*e*, proving its chemical nature as hydroxide anion. The formed hydroxide anion then migrates away from the graphene substrate through the hydrogen bond matrix of liquid water by proton exchange with a neighboring water molecule to propagate to a farther position. To distinguish the observed state against the usually assumed adsorption state of *OOH, we denote the dissociated state observed in the AIMD simulations as *O…OH^δ−^, where the ellipsis “…” represents that the dissociated hydroxide anion is separated from the *O by water layer and the superscript indicates the negative charge on the dissociated OH species. Simultaneously, the left oxygen species at the Fe single sites is well solvated by adjacent water molecules by forming two hydrogen bonds, and we neither observe the dissociation nor protonation of the Fe–O species.

In the traditional associative mechanism, the second PCET step transforms the *OOH species into the adsorbed O species (*O). Note that the *O species is not the same as that in the *O…OH^δ−^ since the catalyst has an overall positive charge in the latter case while the formal is neutral. Figure 1b shows the AIMD snapshots of the *O species exposed to liquid water. Interestingly, the *O species also exist for only a short time as the *OOH species. By uptaking a proton from the adjacent water molecule, the *O is quicky transformed into an adsorbed hydroxyl and a free OH in the liquid water in ~1 ps. We therefore denote the two hydroxyls as *OH…OH^δ−^.

Supplementary Fig. 3b shows the MD snapshots of the adsorbed hydroxyl intermediate (*OH) resulted from the third proton coupled electron transfer step exposed to liquid water. When starting from the intact Fe–OH configuration, unlike the *OOH and *O, the hydroxyl does not go through dissociation during the time scale (~30 ps) of our MD simulations. This can be attributed to the stronger nucleophilicity of oxo species compared to the hydroxyl species on Fe single site, so that the proton transfer from the water molecule to the *OH species is relatively harder than to the *O species as observed in Fig. 1b. However, when a dissociated initial configuration (OH is placed away from the Fe single site) is used for the simulation, the OH species can stay away from the Fe single site which is occupied by a coordinated water molecule (denoted as *OH_2_…OH^δ−^ or *…OH^δ−^). Such phenomenon has origin in the strong stabilizing effect of solvation on the hydroxide ion, so that the transformation between *OH and *…OH states is rather sluggish in both ways. Considering the dissociated configuration in the preceding *OH…OH^δ−^ intermediate (corresponding to the *O in the traditional mechanism), the hydroxide anion would be expected to be kinetically trapped away from the active center throughout the lifetime of the whole reaction event. Finally, the fourth proton coupled electron transfer step leads to the formation of water, which completes the catalytic cycle.

In a primary conclusion, the ORR on FeN_4_ in aqueous medium follows a dissociative mechanism of: (i) * + O_2_ → *O_2_, (ii) *O_2_ + H^+^ + *e*^−^ → *O…OH^δ−^, (iii) *O…OH + H^+^ + *e*^−^ → *OH…OH^δ−^, (iv) *OH…OH^δ−^ + H^+^ + *e*^−^ → *…OH^δ−^ + H_2_O, (v) *…OH^δ−^ + H^+^ + *e*^−^ → * + H_2_O. We would also like to note that the proposed mechanism in this work differs from the traditional dissociative mechanism where the *O_2_ is directly dissociated in the chemisorption or the first PCET step.

Encouraged by the revealed dissociative pathways, we extend our investigation to some other commonly used period 4 transition metal centers (Co, Ni, Cu) by AIMD simulations with explicit solvation on corresponding ORR. The results show that for the first PCET step, no spontaneous transformation from intact *OOH to *O…OH was observed in the timescale of ~30 ps for all three catalysts. However, when the *O…OH is used as the initial configuration for the AIMD simulations, all three catalysts could readily further transform to *OH…**2**OH, while Fe-N_4_/C could not (Fig. 1a). For the second PCET intermediate (*O in the traditional mechanism), all catalysts studied here could spontaneously form the configuration of *OH…OH, and NiN_4_/C and CuN_4_/C could further transform to *…**2**OH in the AIMD simulation (Fig. 1b). For the unobserved desorption of *OH on FeN_4_/C, the transformation of *OH to *…OH is both observed in the NiN_4_/C and CuN_4_/C (Fig. 1c). Notice that these formed configurations, once formed, are stable during the time scale (~30 ps) of our AIMD simulations. These results suggest that the dissociative mechanism and the further generation of hydroxide species could be somewhat a universal phenomenon for transition metal-based SACs in aqueous or other protic media. Since the M-N_4_ center has only one metal site (unlike bulk surfaces), only through the side-on configuration of *O_2_ (di-sigma mode) can the system access the di-oxo configuration where both 2*p*–2*p* $\sigma$ and 2*p*–2*p* $\pi$ bonds in O = O are broken. However, the interaction between Fe 3*d* and O 2*p* is not strong enough so that it favors the end-on configuration instead of the side-on configuration, and it is the same case for other late transition metals such as Co, Ni, and Cu which have even lower *d*-band center energies (Supplementary Fig. 4). To this date, the only M-N_4_ center that we have found to favor the di-oxo configuration is the Mn-N_4_, probably due to a higher *d*-band center of early transition metals.

**Fig. 2:**
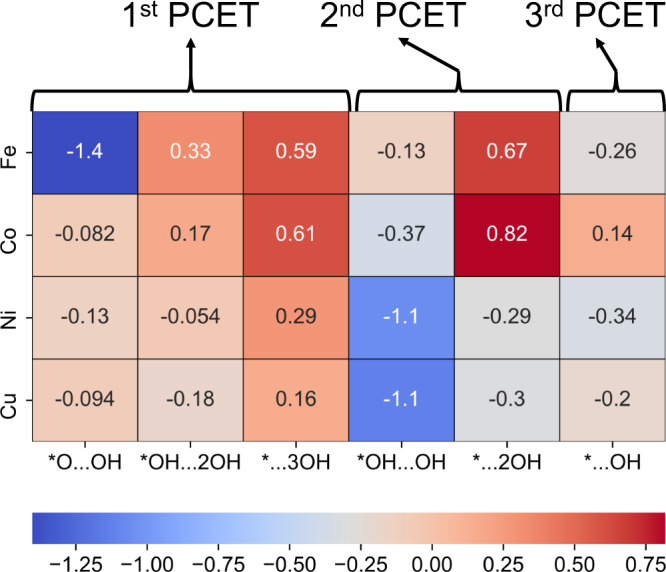
Energy diagram of different reaction intermediates of different transition metal single atom catalyst to form pseudo-adsorption state of OH^δ−^. The heatmap shows the Δ*E* to form each pseudoadsorption state of OH^δ−^. The first PCET step shows the dissociation of *OOH species and the further protonation steps of *O…OH^δ−^ species. Second PCET step shows the protonation steps of *O species. Third PCET step shows the dissolution of *OH species into the solvent environment by the protonation from an adjacent water molecule.

Since the timescale that AIMD can access is somewhat limited, we proceed to investigate the energetics for the formation of dissociated hydroxide on each ORR intermediate through reacting with a neighboring water molecule. The calculated free energy differences are shown in Fig. 2, with the Δ*G* values colored according to a cool–warm colormap. For the *OOH species, the dissociation of O–O bond happened on Fe single atom site has a highly favorable Δ*G* of −1.40 eV, which is consistent with the MD simulation that the *OOH species readily dissociates into a hydroxide anion and a positively charged *O in just ~2 ps. But for the other three metal centers, the dissociation of O–O bond has a less negative Δ*G*, showing a less favorable transformation, which could account for the failure to observe spontaneous dissociation in the AIMD simulation. The variation in O–O dissociation ability is rooted in the electronic structure of the metal center. The partial density of state (pDOS) in Supplementary Fig. 4 shows that the matching between 3*d* states of the metal center and the 2*p* states of O_2_ follows the order of Fe > Co > Ni > Cu, as characterized by the integrated overlapping area, indicating a decrease in the bonding interaction between metal and O_2_. This is also supported by the energy of the *d*-band center of the four metal centers which follow the same order of Fe > Co > Ni > Cu. Since the strong Fe 3*d*–O 2*p* interaction leads to partial filling of the ${\pi }^{* }$state in O–O, the O–O dissociation is more strongly favored for Fe than for Co, Ni, or Cu. Furthermore, we consider whether the left *O species can further capture the proton from the adjacent water molecules to form deeper protonated reaction intermediates and more hydroxide anions, i.e., *O…OH^δ−^ + H_2_O → *OH…**2**OH^δ−^ and *OH…**2**OH^δ−^ + H_2_O → *H_2_O…**3**OH^δ−^. The energy diagram in Fig. 2 shows that it is rather thermodynamically unfavorable for the left *O species to undergo further protonation by the solvent water molecules for Fe-N_4_/C catalyst and thermodynamically favorable to undergo one more protonation for the other three metal catalysts. This can be attributed to the more stable electronic configuration of Fe-oxo species on Fe-N_4_/C catalyst originated in the higher *d*-band center of Fe and the stronger interaction between Fe 3*d* and O 2*p* states as just discussed.

According to the traditional understanding of the ORR process, the formed *O…OH^δ-^ state would experience further proton-coupled electron transfer (PCET) to form water and *O intermediate (i.e., *O…OH^δ−^ + H^+^ + *e*^*−*^ → H_2_O + *O). Based on the AIMD simulation, for all four catalysts the *O intermediate can undergo protonation by uptaking a proton from an adjacent water molecule (i.e., *O + H_2_O → *OH…OH^δ−^). This process is calculated to be thermodynamically favorable (Fig. 2). The formed *OH…OH^δ−^ cannot undergo further protonation (i.e., *OH…OH^δ−^ + H_2_O → *H_2_O…**2**OH^δ−^) by another water molecule until the next PCET to form the *OH intermediate (i.e., *OH…OH^δ−^ + H^+^ + *e*^*−*^ → H_2_O + *OH) for Fe-N_4_/C and Co-N_4_/C catalysts. After that, this *OH intermediate can readily uptake a proton from neighboring water to form *…OH^δ−^. Note that due to the high computational cost from the explicit solvation and sheer size of the system, free energy profiles are not obtained for systems shown in Fig. 2 except for Fe-N_4_/C on which we will elaborate in the next section. Nonetheless, the above thermodynamics and AIMD simulations could provide sufficient evidence that it is thermodynamically and kinetically favorable for each electrochemical intermediate to transform into a state with one (or several) dissociated hydroxide species that is well solvated by water environment and separated from the catalytic center.

### Mechanistic nature of the pseudo-adsorption state

**Fig. 3:**
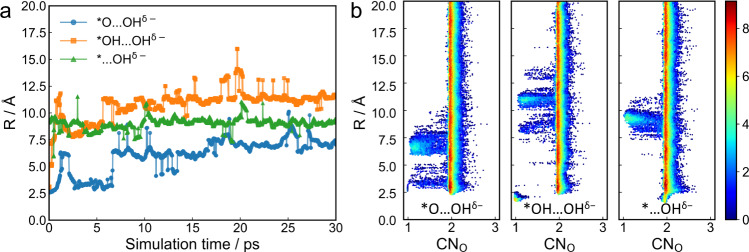
Spatial evolution and equilibrium distribution of the pseudo-adsorption species from AIMD. **a** The *z* coordinate of the O in dissociated OH^δ−^ relative to that of the Fe single site (denoted as R) in *O…OH^δ−^, *OH…OH^δ−^, and *…OH^δ−^ intermediates during the 30 ps AIMD simulations. **b** Probability density distribution of CN_O_ as a function of R, from the three equilibrated AIMD trajectories. The scatter markers are colored by the frequency of occurrence (in logarithm scale) according to the color bar on the right. CN_O_ denotes the coordination number of O by H.

The previously presented AIMD results show yet another puzzling behavior of the reaction intermediates: the dissociated hydroxide species does not keep migrating away from the active center as we would expect to happen to a regular free-moving hydroxide, but it would be somewhat confined at the electrochemical interface and within a certain distance from the Fe single site after equilibration, which we denote as a “pseudo-adsorption” state. In the following sections, we focus on the Fe-N_4_/C catalyst, which is one of best-performing ORR systems and a prototypical model SAC, to explore the mechanistic nature of the pseudo-adsorption state. Figure 3a shows the *z* distance of the confined hydroxide species relative to the Fe single site, denoted as R, in different reaction intermediates during the 30 ps AIMD simulations. The spikes correspond to the fast proton exchange process between the dissociated hydroxide and neighboring water molecules with timescale of <0.1 ps. Therefore, the hydroxide species is not a static spectator, but is in constant proton exchange with its solvation shell. In all of the three trajectories, the dissociated hydroxide can respond to the redox events at the catalytic center by diffusing toward or away from the catalyst surface, reaching the equilibrium distance within ~5 ps and persists thereafter. More insights into the behavior of such pseudo-adsorbed hydroxide could be gained in the probability density distribution plot of coordination number of O by H (CN_O_) versus R in Fig. 3b. The bright regions at CN_O_ = 1 corresponds to hydroxide species, and the equilibrium distances are ca. 7, 11, and 9 Å from the catalyst surface for *O…OH^δ−^, *OH…OH^δ-^, and *…OH^δ−^, respectively, which corresponds approximately to the height of the second, fourth, and third water layer above the catalyst surface. The long tail connecting the regions with CN_O_ = 1 (hydroxide) and CN_O_ = 2 (water) further evidences the frequent proton exchange between the dissociated hydroxide and its hydration shell, and by so the hydroxide could access a neighboring water layer. We could see some other minor CN_O_ = 1 spikes, such as the *R* = 3 Å region in *O…OH^δ−^ and the *R* = 8 Å region in *OH…OH^δ−^, suggesting that they are also local minima on the free energy surface that are thermally accessible at 300 K. However, the relative energy difference among those minima shifts upon change of the reaction intermediate on FeN_4_ center, and the hydroxide can always quickly equilibrate within 1 ps and stabilize around the new global minimum position.

**Fig. 4:**
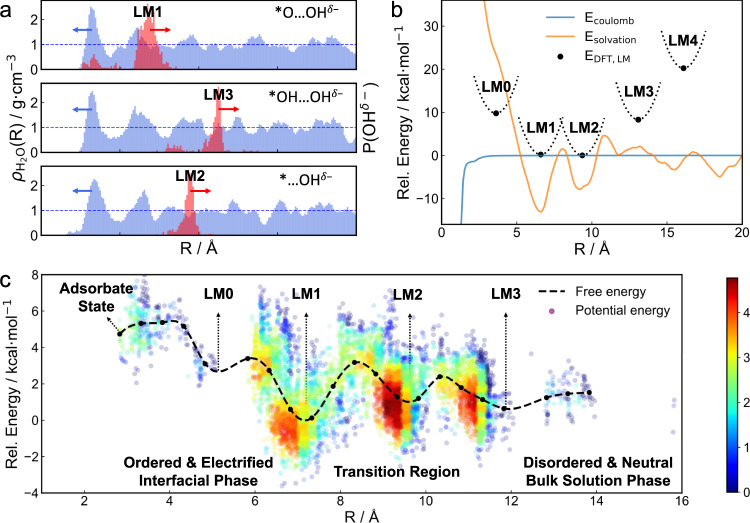
Analysis of the origin of the pseudo-adsorption state. **a** The water density distribution ${\rho }_{{{{{{{\rm{H}}}}}}}_{2}{{{{{\rm{O}}}}}}}$ and the probability density P(OH^δ−^) of pseudo-adsorbed OH^δ−^ in systems of *O…OH^δ−^, *OH…OH^δ−,^ and *…OH^δ−^, from equilibrated AIMD trajectories. **b** Thermodynamic insight into the pseudo-adsorption state. **c** Approximated free energy profile of the pseudo-adsorbed hydroxide based on AIMD trajectories, obtained by integrating out the degrees of freedom other than R. The AIMD data points are colored by frequency of occurrence (in logarithm scale) according to the color bar on the right. The “LM” denotes the local minima by performing geometry optimization on selected configuration with the pseudo-adsorbed hydroxide at different R.

To gain insight into this intriguing pseudo-adsorption behavior, we first obtain the local water density ${\rho }_{{{{{{{\rm{H}}}}}}}_{2}{{{{{\rm{O}}}}}}}$ as a function of R based on effective counting within the region of 1.67 < CN_O_ < 3 for each subplot in Fig. 3b. We have also obtained the density distribution of the pseudo-adsorbed hydroxide species P(OH^δ−^) based on effective counting within the region of CN_O_ < 1.67 and *R* > 2.5 Å (to exclude the *OH on Fe center) for each subplot in Fig. 3b. The water density and density distribution function of pseudo-adsorbed hydroxide are overlapped in Fig. 4a. The water density function suggests water accumulation near the catalyst surface that leads to significantly higher water density than in the bulk water layers. This could be attributed to the polarization of the interfacial water by O-containing reaction intermediate, resulting in a more rigid local solvation network, as is characterized by the sharper peaks corresponding to the first and second water layer above the catalyst surface (contact bilayer). Beyond the 3rd or 4th water layer, the water density gradually converges to the flatter bulk-like distribution around 1 g cm^−3^, marking the end of the polarized interfacial region. It is also notable that the peak positions of the P(OH^δ−^) are not overlapping with the peak positions of ${\rho }_{{{{{{{\rm{H}}}}}}}_{2}{{{{{\rm{O}}}}}}}$, indicating the equilibrium position of pseudo-adsorbed hydroxide to be in-between the neighboring water layers rather than inside a water layer.

To further investigate the origin of the equilibrium position of the pseudo-adsorption state, we obtained five putative local minima (LMs) by performing geometry optimization on selected configurations of the *…OH^δ−^ system with different R. The datapoints of LM0–5 are plotted as black spheres in Fig. 4b, together with a dotted parabola curve that represents the local potential energy landscape around each LM. It can be seen that, LM1 and LM2 with the lowest energy are located at 6.6 and 9.4 Å, respectively, which corresponds to the transitioning between the direct contact catalyst/water interface and the bulk solution. Since the water density distribution differs greatly in such two regions, we would expect the solvation stabilization of hydroxide to also differ accordingly as R increases. Here we plot the solvation energy *E*_solvation_ based on the assumption that the solvation energy is proportional to the local water density distribution (details in the “Methods” section) in Fig. 4b as the orange curve relative to the hydration energy of hydroxide ${E}_{{{{{{\rm{solvation}}}}}}}^{\ominus}$ reported in ref. ^29^ In the region of *R* < 5 Å, the solvation effect is weak due to zero water density at the catalyst surface. In the region of 5 Å < *R* < 10 Å, we could see the formation of two minima due to a higher local water density than in the bulk solution, contributed by the water accumulation layers below at ca. *R* = 3.5 and 6.5 Å. The R position of these two minima are in line with the DFT-calculated LM1 and LM2. Beyond *R* = 10 Å, the *E*_solvation_ converges to the bulk behavior, with flat periodic pattern and shallow minima. Since the catalytic center has a partial positive charge and the pseudo adsorbed hydroxide is negatively charge, we would expect an electrostatic interaction between them.

The calculated electric potential energy according to Coulomb’s Law (details in the “Methods” section) *E*_coulomb_ (blue curve in Fig. 4b) flattens out beyond 3.0 Å due to the inverse proportionality to R, with its variation decayed to within 1 kcal/mol. Hence, we could conclude that the stabilization of pseudo-adsorbed hydroxide is mainly a result of solvation instead of electrostatics. We would also like to note that the *E*_coulomb_ model only holds beyond the covalent bond region where the classical electrostatics apply, and that the analytical *E*_solvation_ fails to capture the first LM since it does not consider many aspects of a realistic solution such as configurational entropy. Nevertheless, this analysis could provide qualitative insights into the potential energetic trends and the relative contribution of different interactions at the interface–bulk transitioning region.

Based on the AIMD simulations, we construct an approximate free energy profile of the system along R by integrating out the degrees of freedom other than R. In Fig. 4c, we show the calculated free energy profile in dashed curve and the datapoints from AIMD as spheres colored by frequency of occurrence in logarithm scale. The free energy landscape is surprisingly flat along R, with most barrier heights within 3 kcal/mol. This could explain the thermal accessibility of neighboring minima at 300 K and the fast equilibration of the hydroxide to its new global minimum (GM) position within 1 ps as the reaction proceeds and the adsorbate at Fe center changes. The near-surface region corresponding to the adsorbate state is ca. 5 kcal/mol higher than the GM, which is due to a lack of solvation stabilization, and it drives and keeps the hydroxide away from the catalyst surface. A few minima LM0–3 can be observed at *R* = 5.1, 7.2, 9.7, and 12.0 Å, respectively, with the free energy eventually flattening out to ca. 1.0 kcal/mol beyond LM3, which is the solvated state in bulk solution phase. The shape of the free energy profile resembles that of the *E*_solvation_ curve in Fig. 4b in the range of 6 Å < *R* < 11 Å, which again evidences the major role of solvation stabilization in the dynamic confinement of the pseudo-adsorbed hydroxide. The overestimation of energy of LM3 and LM4 (minima on the DFT potential energy surface instead of the free energy surface) in Fig. 4b can be attributed to the lack of configurational entropies which favors the more disordered bulk solution phase.

Note that we are aware of the inconsistency in sampling density along R, which leads to inaccuracy in free energy evaluation in the sparsely sampled regions due to configurational bias. A metadynamics simulation might provide better sampling, however, we could not find a proper analytical collected variable for R which can be evaluated on the fly. In addition, the barrier heights and well depths are found to be within a few kcal/mol, which is a rather sensitive energy scale that most methods could not quantitatively describe. Hence, we expect reordering of the relative energy of the first few GMs as the adsorbate varies, although not investigated herein.

The free energy landscape of the pseudo-adsorbed hydroxide is highly dependent on the chemical nature of adsorbates at the catalyst surface. To be specific, the size of the interfacial region, where the water layers are more polarized and rigid, differs among systems with different adsorbates. H-bond interaction stabilizes the states with higher local water density, while the configurational entropy stabilizes the states with more disordered and chaotic water arrangements. Such two contributions compete with each other and create a GM at the crossover region between interfacial water phase and bulk solution phase. In *…OH^δ−^ system, the adsorbate at Fe center is the charge-neutral dynamically coordinated water (the local maxima at CN_O_ = 2 and *R* = 2.5 Å in Fig. 3b right panel, and the spike at *R* = 2.5 Å in Fig. 4a lower panel), and the GM of the free energy profile is located at LM2. However, in *OH…OH^δ−^ system, the adsorbate at Fe center is *OH which has a larger dipole compared to water, causing a stronger polarization of the water molecules in the contact bilayer. In addition, *OH is a highly directional H-bond donor and acceptor, which causes a more rigid ordered local H-bond network compared to the bulk solution phase. As a result, the interfacial region is extended and the GM shifts to LM3. In sharp contrast, the *O adsorbate in *O…OH^δ−^ system has no directional preference in H-bond formation, which lead to an enhanced disorder within the contact bilayer. Consequently, the interfacial region goes into an early end, right beyond the second water layer, and the GM shift to a closer-to-surface position at LM1. The link between free energy and local water density distribution would not be gained without a sufficiently thick water slab (to properly describe the bulk solution behavior ~10 Å beyond the catalyst surface) or with continuum-based implicit solvation model which provides no statistical information on the water dynamics at all.

To gain quantitative insights into the potential of the surface in the AIMD simulations, we calculated the work function of the reaction intermediates (Supplementary Table 1). The SHE-scale work function of *O…OH^δ−^, *OH…OH^δ−^, and *…OH^δ−^ are calculated to be 0.47, 0.36, and 0.13 V, respectively, which correspond to the range of moderate-to-high overpotential range for ORR. At even higher overpotentials, the interfacial phase will be extended to an even farther position beyond LM3, with a steeper downhill free energy. However, it is not clear at this point whether the hydroxide species will be driven into the bulk solution phase faster since the reaction coordinate is also extended. If the kinetics is favorable enough, every generated pseudo-adsorbed hydroxide will migrate in time to the bulk solution phase, otherwise there could be an accumulation of hydroxide species at the transition region, especially for alkaline ORR.

In terms of pH condition, the above simulations correspond to a near-neutral or alkaline media where water molecules work as the major proton source and the concentration of hydronium is negligible. In acidic media, the higher concentration of hydronium could further facilitate the outward migration of hydroxide by creating a concentration gradient, and it would also alleviate the hydroxide accumulation problem if there were any. In very acidic media, the lifetime and population of pseudo-adsorbed hydroxide would be significantly reduced to negligible, but we then would expect a similar spike in the water density distribution function in the transition region (since water is to acidic ORR as hydroxide is to alkaline ORR, as the deprotonated form of the proton source species).

At a realistic electrochemical interface in operation, there will be a higher electron density at the catalyst surface (due to higher overpotential needed for a higher current density) and also cations in the electric double layer (EDL). To evaluate how such complexity will influence the behavior of the pseudo-adsorbed hydroxide, we further set up a more realistic model by introducing sodium atom into the contact bilayer. After the redistribution of electron density by self-consistent field iteration in DFT calculation, the sodium atoms becomes a cation and the extra electron density get localized on the catalyst surface, establishing an outer Helmholtz plane under the periodic boundary condition^30,31^. The work function of the surface is brought from 0.47 V to a more negative value of 0.25 V in SHE scale (Supplementary Table 1), which corresponds to a high overpotential and current density scenario in a practical device. Under this approximate EDL setup, we are still able to observe the formation of pseudo-adsorption state in *…OH^δ−^ system during the AIMD simulation. The distribution function indicates that the hydroxide anion is equilibrated at ~11 Å away from the catalyst surface (Supplementary Fig. 5). The new equilibrium position corresponds to the LM3 in Fig. 4c and while the previous result is at LM2. This can be attributed to the fact that the electrified surface and the presence of cations in the EDL will enhance the polarization effects to extend the interfacial water phase to a farther position. In a realistic electrochemical cell with a much higher applied potential, the extension of interfacial region could be even more pronounced and could reshape the free energy landscape to form more LMs beyond LM3, which we will discuss further in the last section.

### Dynamic coordination of water to the active center

**Fig. 5:**
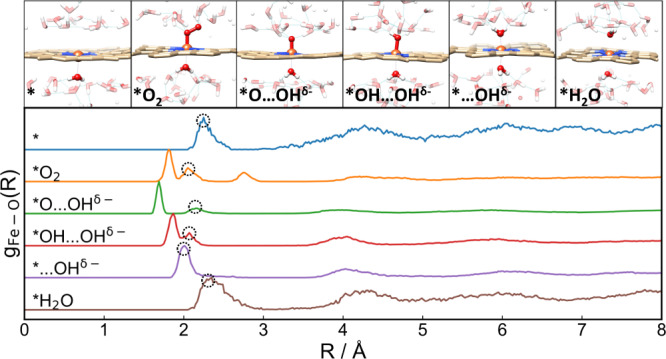
The distribution of the backside axial water. The radial distribution function *g*(*r*) for the investigated reaction intermediates with their representative AIMD snapshots labeled on the top.

The explicit water environment affects the ORR chemistry not only by stabilizing dissociated hydroxide species into the special interfacial state of pseudo-adsorption but also via direct coordination to the catalytic center in a more local fashion. In all of the AIMD simulations we performed, a backside (relative to the frontside where ORR takes place) Fe–OH_2_ coordination is observed after equilibration. Note that such configuration is realistic since the M–N_*x*_/C catalysts are usually engineered to be nanoporous to enhance the specific surface area, hence water molecules could access both sides of the graphitic carbon layers without suffering much hindrance. The representative AIMD snapshots and Fe–O radial distribution functions of the investigated reaction intermediates are summarized in Fig. 5. It can be seen that although the backside water ligand adopts the same axial position in all intermediates, the Fe–O_axial_ bond lengths vary (O_axial_ stands for the O in the backside axial water ligand, marked by dotted circle in Fig. 5). In the initial and final state, the Fe–O is 2.26 Å long, which is significant longer than that in a typical Fe(H_2_O)_6_ complex (2.00 Å). Upon the chemisorption of O_2_, the Fe–O_aq_ is shortened to 2.06 Å due to the formation of a more positively charged Fe center after it transfers an electron to activate molecular oxygen into peroxide species. In *O…OH^δ−^, the formation of Fe=O leads to partial coordinative saturation and weakens the backside Fe–O_axial_ to lengthen to 2.16 Å. Upon the following two PCETs to form Fe–OH and Fe–OH_2_ at the front side, the frontside Fe–O gradually weakens and the backside coordination strengthens as characterized by the shortening of the Fe–O_axial_ to 2.07 and 2.02 Å, respectively. Note that although the Fe single site in *…OH and * has the same coordination geometry (with axial water ligand on both sides), they feature distinct oxidation states, with the latter being more positively charged which leads to stronger binding to the electron-donating O in water ligand. It can be seen that on different reaction intermediates, the backside axial water ligand binds to the Fe single site dynamically and adapts to the different electronic structure at the active center induced by different frontside species.

**Fig. 6:**
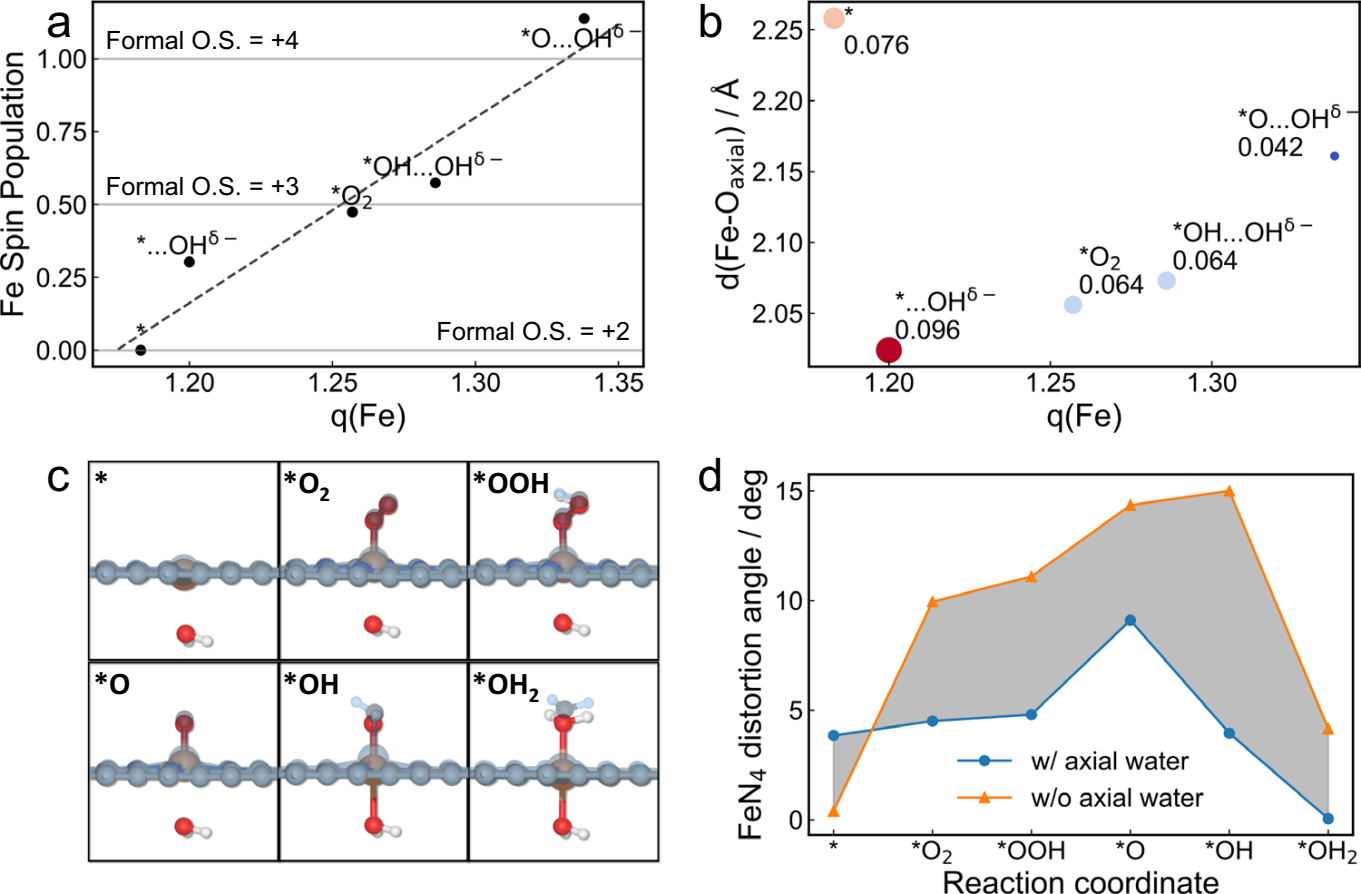
Effects on the catalytic center induced by the backside axial water. **a** The scatter plot of Mulliken spin population versus the Bader charges on Fe along the reaction coordinate. The fitted linear correlation is shown in black dashed line, and the formal oxidation states of Fe are marked by gray lines. **b** The scatter plot of Fe–O_axial_ distances versus the Fe Bader charges. The charge transfer from backside axial water is labeled next to each data point. **c** Overlapped geometries of gas phase reaction intermediates with (colored) or without (transparent blue) backside axial water ligand. **d** The evolution of FeN_4_ distortion angle along the reaction coordinate for gas phase reaction intermediates with(blue lines) or without(orange lines) backside axial water ligand. The shaded area represents the difference of FeN_4_ distortion angle in the two cases.

To investigate the effect of the dynamic water coordination on the electronic structure of the Fe center, Bader analysis is performed (tabulated in Supplementary Table 2). In Fig. 6a, the Bader charges of the Fe center is plotted against the Mulliken spin population assigned based on the coordination state of each reaction intermediates. Since the initial state Fe(II) has zero spin, the Mulliken spin population of 0, 1/2, and 2/2 would correspond to formal oxidation state (OS) of +2, +3, and +4, respectively. Note that the *S* = 2/2 for Fe(IV) here also indicate the critical role of backside axial water in altering the crystal field of Fe to an octahedral pattern, since the square planar pattern would predict *S* = 0 for Fe(IV) otherwise (Supplementary Fig. 6 and Supplementary Table 3). Surprisingly, despite that a relationship can be observed that a more positive Bader charge corresponds to higher spin and formal oxidation states, the data point from *…OH^δ−^ and *OH…OH^δ−^ deviate from the trend and lead to bad correlation (*R*^2^ = 0.92). Such deviation is a result of the charge transfer from backside water coordination. In Fig. 6b we plot the Fe–O_axial_ bond length versus the Bader charge of Fe and annotate next to each marker its corresponding charge transfer value from backside axial water to the Fe center. It is somewhat surprising that despite the seemingly small value of the water-to-Fe charge transfer, it contributes to ~20% of the variation in Bader charge of Fe center along the reaction coordinate. It can also be concluded roughly that a shorter Fe–O_axial_ distance leads to a larger charge transfer. However, it is not always the case since there are multiple variables (Fe oxidation state, nature of the frontside ligand and crystal field), and none of them can solely determine the amount of such charge transfer. The geometric distortion may be playing the most decisive role here. By resorting to a simplified gas phase model with or without backside axial water ligand, we observe a geometric difference in the FeN_4_ moiety after introducing the backside axial water (Fig. 6c). For example, in the *OH intermediate without backside axial water, the Fe center is dragged out of the carbon matrix plane by the hydroxyl dramatically to form a square pyramidal configuration, while in the *OH with backside axial water the Fe is relatively kept in plane in a slightly distorted octahedral configuration. By calculating the distortion angle of the FeN_4_ from the carbon matrix plane, it is shown clearly in Fig. 6d that the presence of backside axial water could counteract the geometric distortion of the Fe-N_4_ center induced by the frontside species while avoiding drastically changing the coordination states as strong field ligands could do^32^. Through forcing the coordination field to an octahedral field, the binding strength of ORR intermediates on the Fe center is weakened, which alleviates the over-binding problem on Fe single sites. Supplementary Fig. 7 shows that including the backside axial water could solely reduce the ORR overpotential by 0.06 V via making the *O_2_ to *OOH step less endothermic. The binding modulation strongly resembles the role of axial ligands in the Heme motif^33^, and the similar effects of evolving solvation/adsorbate configuration on reactivity of the catalytic site has also been reported^34^. By alleviating the distortion and stabilizing the octahedral configuration, the Fe-N_4_ moiety is less prone to integration through demetallation under a high reduction potential^35^, which is the major source of destabilization of the FeN_4_/C catalysts in long term operation^36^. In fact there have been reports where stability is enhanced through constructing backside coordination sites to form a similar Fe-N_5_ moiety^37,38^. It is also possible that the CN = 5 signal of Fe probed by *operando* XAS in these studies are partially contributed by the backside axial water ligand, especially for the ones with porous nanostructure that is highly water-permeable. We note here that this part of the comparative investigation is based on the traditional mechanism in gas phase since some control group models spontaneously destabilized when explicit solvation is considered, but it could well reflect the effect of backside axial water coordination.

The deeper implication is that the backside axial ligand adapts to different Fe oxidation states and nature of the frontside ligand, thereby affecting the binding strengths of different reaction intermediates in different ways and extents. This makes possible bypassing the linear free energy relationships which is usually based on static models and without explicit solvation^39^. Viable approaches may include engineering the local wettability near the active site in the material or introducing an appropriate amount of other redox-poor coordination sites, such as halides or sublayer dopants, into the reaction system to artificially construct such axial coordination configurations.

### Revised reaction mechanisms

**Fig. 7:**
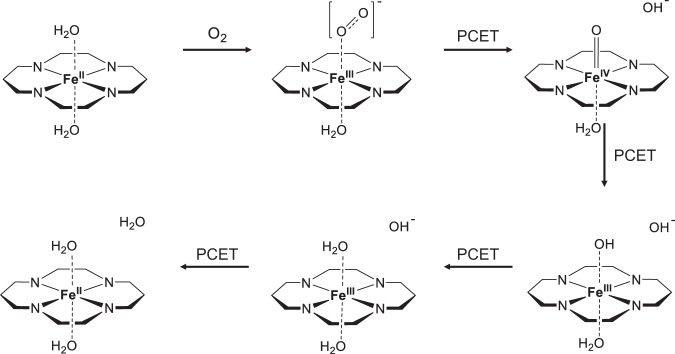
Revised mechanism of ORR on FeN_4_ in aqueous medium. For the convenience of view, the N-doped nanocarbon support is simplified into a simple skeleton structure.

Based on the above analysis, we could summarize the revised mechanism of ORR on FeN_4_ in aqueous medium (Fig. 7): (1) In the initial state, the Fe is a Fe (II) species bound to the pyrrolic N_4_ site. (2) Upon binding a molecular oxygen, one electron is transferred from Fe d orbital to O_2_ ${\pi }^{* }$ orbital to weaken the O=O bond. The Fe (II) is hence oxidized to Fe (III) while the O_2_ becomes [O_2_]^−^. (3) In the first PCET step, both ET and PT take place at the terminal O to form a transient *OOH with lifetime of ~2 ps. The *OOH then spontaneously dissociates, with the O–O broken heterolytically, to form a pseudo-adsorbed hydroxide anion and a positively charged *O where Fe is pushed to a higher IV oxidation state through forming an iron-oxo species. Meanwhile, the pseudo-adsorbed hydroxide diffuses to the transition region between the electrified interface and bulk solution and equilibrates at the GM of the free energy surface dictated by solvation stabilization. (4) In the second PCET step, the oxo is protonated to hydroxyl, and the Fe (IV) is reduced to Fe (III). (5) In the third PCET step, both the PT and ET take place at the hydroxyl to form a water molecule. (6) In the final PCET step, the pseudo-adsorbed hydroxide undergoes PT while the Fe (III) center undergoes ET to return to the initial Fe (II) state. We argue that the two processes could be considered coupled since the pseudo-adsorbed hydroxide could “feel” the changes in the electronic structures at the active center, with just two or three H-bond-bridged water molecules in between and could respond accordingly by diffusing to a closer/farther position or undergoing protonation. The last step is rather essential to the whole mechanism since Fe (III) can over-bind water, and only after its reduction to Fe (II) can it enter the next catalytic cycle. In all reaction intermediates, a water molecule loosely coordinates to the Fe single site in the backside axial position, which dynamically changes the binding strength and bond length to adapt to different reaction intermediates and promote the catalytic center to adopt an octahedral coordination configuration. Such geometric effect not only prevents the FeN_4_ moiety from disintegration or demetallation but also overall weakens the binding of the reaction intermediates to avoid the “deep well” in the free energy profile from over-binding which is usually seen on pyrrole-type Fe-N_4_ moiety^40^.

To assess the energetics of the revised ORR mechanism, we constructed the free energy diagram based on intermediate geometries from simulated annealing and the CHE model, as is shown in Supplementary Fig. 8 (detailed energies summarized in Supplementary Tables 4 and 5). Compare to the gas phase case, the chemisorption of O_2_ is stronger in liquid phase probably due to the hydrogen bonding stabilization of the anionic peroxide species *O_2_, which is consistent with previous studies^27^ In the first PCET step, which is also the rate determining step (RDS) in the traditional mechanism, the formation of pseudo-adsorption state lowers the energy of the whole system significantly since the dissociated *O and OH^δ−^ together are subject to stronger solvation effect compared to an intact *OOH species. Thus, the RDS in liquid phase is no longer the protonation of *O_2_ to *OOH but the last PCET step where the pseudo-adsorbed hydroxide is finally protonated while the catalytic center gets reduced. The approximated thermodynamic overpotentials are calculated to be 0.76, 0.70, and 0.61 V for gas phase model, gas phase model with backside axial water, and the liquid phase model, respectively. The comparison demonstrates that the lack of explicit water environment and solvation effect could be a factor contributing to the overestimation of thermodynamic ORR overpotential (experimental: 0.35–0.45 V, depending on synthetic protocol and metal loading) under the CHE scheme^41–43^.

Zooming out to inspect the whole electrochemical interface in a larger spatial scale, we propose that the pseudo-adsorption state of reaction intermediates could indicate the presence of an extended active region where some reaction steps take place not necessarily at the direct catalyst surface but at the pseudo-adsorbed species that is confined in the interfacial region. Since such region is located between the direct surface of the catalyst and the bulk phase of the solvent, we dub it as “bulk interface”. In our case of ORR on FeN_4_ in aqueous medium, after the first PCET, the dissociated hydroxide leaves the direct catalyst surface and get confined in the bulk interface region which is ~10 Å away from the catalytic center. Such confined “pseudo-adsorption” state and the catalytic center are spatially separated but connected via hydrogen bond network so that the pseudo-adsorbed species could respond to the redox event at the catalytic center. In the PCET steps, the proton species (in form of hydronium in neutral/acidic media or water in alkaline medium), which diffuses from the anode to the cathode through the proton exchange membrane, protonates the pseudo-adsorbed hydroxide in the bulk interface without needing to travel to the direct surface of the FeN_4_/C catalyst. In other words, the whole bulk interface plus the catalyst constitutes an extended reaction system due to the long-range interfacial behavior. Therefore, the overall ORR process would be less subject to mass transfer limitations and could show an accelerated reaction kinetics.

The above discussion assumes that the positively charged surface species (e.g., *O in *O…OH^δ−^) resulted from the release of hydroxide anion could maintain its net charge until the final PCET step. Since at this point it is unclear to which extent the PT and ET are coupled, i.e., whether the applied reduction potential at the cathode would reduce the catalytic center back to the initial charge state before protonation events could take place, we further consider the following cases. If the charge transfer at electrode were to take place in a shorter time scale compared to proton exchange through the hydrogen bond network, the system would be semi-vertically supplied with an electron by the cathode before solvation configurations could fully equilibrate. We simulate the behaviors of this charged interface by injecting an extra electron into the system. The Bader charge analysis indicates that most of the extra electron density gets located at the catalyst surface to compensate the electron loss from O_2_ activation. It can be seen in Supplementary Fig. 9 that this extra charge causes a more strongly polarized contact bilayer and extends the interfacial region to even further, driving the pseudo-adsorbed hydroxide all the way to an equilibrium position of ca. *R* = 15 Å, which is about the LM4 marked in Fig. 4 and is almost into the bulk solution phase. As a result, the protonation of the hydroxide is less coupled to the redox events at the catalytic center due to the elongated reaction coordinate. The pseudo-adsorption state would be able to diffuse away into the bulk solution to get protonated if there were no box boundary or if the catalyst surface were charged even more (corresponding to a more negative applied potential). However, if that happens, the reduced *O would spontaneously react with an adjacent water to form *OH…OH^δ−^ in a few ps, releasing a hydroxide anion which then diffuses to the bulk-interface transition region to regenerate the pseudo-adsorption state. If the proton exchange were faster than charge transfer kinetics, the pseudo-adsorbed hydroxide anion would be first consumed through protonation. Then the following ET reduces the catalytic center to *O, which similarly regenerates the pseudo-adsorbed hydroxide. In the latter two cases, the overall ORR kinetics would be much less capped by mass transfer since the PCET steps from 1 to 3 all involve the pseudo-adsorption state which is distributed in an extended spatial region and is more accessible to proton exchanging. A rigorous investigation into the interfacial process would require (i) direct evaluation of charge transfer kinetics at the cathode in the presence of explicit solvation (including explicit hydronium or hydroxide to model acidic or alkaline media), (ii) more efficient and extensive configurational sampling techniques, (iii) grand canonical DFT treatment that gives constant-potential energetics (under the current constant-charge scheme, the work function varies along the reaction coordinate)^44–46^, and (iv) multi-reference methods to handle the strongly correlated characteristics of the FeN_4_ with oxygen adsorbates, which we are working on but is beyond the scope of this study.

## Discussion

In summary, we revisit the ORR on a series of metal single atom catalyst in aqueous medium by performing ab initio molecular dynamics (AIMD) with explicit solvation. Under the effect of explicit water, a dissociative mechanism is identified and shows a distinct free energy profile compared to the traditional mechanism with improved agreement with experiments. It is demonstrated that a hydroxide species is dynamically confined in the interfacial region in a “pseudo-adsorption” state at about 10 Å away from the active site, originating from the balance between the H-bond interaction contribution and the configurational entropy contribution in the solvation free energy. Such confined “pseudo-adsorption” state is dynamically confined in one of the shallow, thermal-accessible local minima on the flat free energy surface dominated by solvation, so that the pseudo-adsorbed species is long-range coupled to the redox event at the catalytic center within timescale <1 ps. Moreover, solvent water can dynamically coordinate to the metal center in the backside axial position, adapting the binding strength and coordination configuration as the reaction proceeds, to module the ORR energetics and structural stability of the M-N_4_ moiety. Thermodynamics calculations and AIMD simulations suggest such phenomenon to be likely universal for other transition metal-based SAC systems. Our study emphasizes the necessity to include realistic aspects such as explicit solvation when modeling electrocatalytic reactions, and the rich chemistry and dynamics that is made possible to capture.

Understanding and harnessing the dynamic behaviors at different spatial scales in the electrochemical interface could unlock a new dimension for catalyst/reactor design and optimization. On the experimental side, the transient lifetime and confined spatial distribution of the “pseudo-adsorption” state makes it intrinsically challenging to directly probe. Moreover, the similarity of its vibrational modes with free hydroxide anion and O–H in water molecules could lead to difficulties in discerning the spectroscopic signals of the “pseudo-adsorbed” hydroxide species from the solvent background. One of the possible direct approaches would be a combination of isotopic labeling and *operando* surface-enhanced spectroscopic methods. Another indirect approach would be *operando* Mössbauer spectroscopy with sufficient time resolution to probe the high spin *S* = 2/2 signal of Fe(IV) which is a signature species in the mechanism that we propose compared to the traditional one. In fact, we believe our revised ORR mechanism could be a partial contributor to the high spin signal probed in FeN_4_–C in some recent reports^8,47^.

Other realistic aspects may also affect the ORR process as discussed. For example, the presence of EDLs could help maintain the pseudo-adsorption state, so that the PT-limiting mechanism may be favored since the hydroxide is kinetically trapped. In the contrary, nuclear quantum effects, which have been reported to significantly enhance the tunneling of proton through the hydrogen bond network and the autoionization of water at the electrode/liquid interface^48^, could facilitate the proton exchange at the interface, leading to the ET-limiting mechanism as well as strengthening the coupling between the catalytic center and the pseudo-adsorbed species. We are currently working on to incorporate the mentioned effects into calculation to establish a unifying realistic model for electrocatalytic interfaces.

## Methods

### DFT settings of static and dynamic calculations

The static calculations were performed by using spin-polarized DFT as implemented in Vienna ab initio simulation package (VASP) code^49–51^. The generalized gradient approximation method with PBE functional was used to describe the electronic exchange and correlation^52^. The plane wave cutoff was set to be 400 eV, and spin polarization was turned on. Fermi smearing was used with a smearing width of 0.1 eV. All atoms were allowed to relax during geometry optimization. Bader charge analysis is performed using the code developed by Henkelman group^53^.

The dynamic calculations was performed using the CP2K package (version 6.6.1), based on PBE functional and a hybrid Gaussian/Plane-Wave scheme (GPW)^54^. The simulations were sampled by the canonical (NVT) ensemble employing Nose–Hoover thermostats with a time step of 1.0 fs at a finite temperature of 300 K^55,56^ for more than 30 ps. The convergence of total energy of the system and the RDF of water (Supplementary Fig. 2) were used as the criteria for equilibration. The GTH pseudopotentials^57,58^ were chosen to describe the core electrons. The wave functions were expanded in optimized double-ζ Gaussian basis sets^59^ and the plane waves were expanded with a cutoff energy of 400 Rydberg. Dispersion correction was applied in all calculations with the DFT-D3 method^60,61^. Dipole correction was also applied throughout to avoid fictious charge interaction between neighboring images under the periodic boundary condition.

We have also performed test AIMD run using SCAN meta-GGA functional on *O…OH^δ−^ system, and the results (Supplementary Figs. 10 and 11) are consistent with the PBE(D3) results. Although we could not afford hybrid functional in AIMD due to the large system size (>1000 atoms), we have computed the reaction free energies using PBE0 and HSE06, and the resulting trend (Supplementary Table 6) is consistent with those using PBE(D3). Therefore, we conclude that the supercell size and functional employed in this study are sufficient for describing the system of study.

### Model set-up of the electrochemical interface

The Fe-N_4_/C catalyst was modeled by a layer of graphene with an embedded Fe-N_4_ moiety. The slab was composed by 6 × 4 graphene supercell in a three-dimensional box (Supplementary Fig. 1) with periodic boundary conditions. The box size was chosen to be 17.04 × 14.76 × 40.00 Å^3^. Besides the catalyst substrate, the vacuum region in the box was filled by aqueous bulk water, which contained 310 H_2_O molecules and possessed an average density of ~1 g/cm^3^. We have also tested a larger 7 × 6 supercell with box size of 21.30 × 17.22 × 40.00 Å^3^ and a non-periodic model, and consistent results of pseudo-adsorption state formation were obtained (Supplementary Figs.12 and 13). Due to difficulty in continuously tuning pH and in explicitly calculating proton chemical potential (a common issue for explicit solvation models)^44^, we focus on near-neutral or alkaline media where the major proton source is water molecules, which can be modeled by the pure water environment (without addition or removal of H)^62^. To give a reasonable initial configuration for the AIMD simulation, each reaction intermediate was pre-optimized before performing AIMD simulations. The other three catalysts were modeled in the same way.

### Calculation of ORR energetics

The free energy calculations for the ORR process is referenced to the computational hydrogen electrode model developed by Nørskov et al. ^22^ with a equation expressed as Δ*G* = Δ*E* + Δ*ZPE*−*T*Δ*S* + *qU* + Δ*G*_PH_, where Δ*E*, Δ*ZPE*, *T*Δ*S*, *qU*, and Δ*G*_PH_ denote the reaction energy, zero-point energy correction; the harmonic entropy contribution; the free energy contribution from the applied potential and the free energy correction from pH, respectively. The rate determining step (RDS) is obtained based on the previous model^22^, where the electrochemical barrier scales with the free energy difference, and a similar prefactor is assumed among surface PCET steps. In this case, the RDS (by kinetics) is also the potential determining step (by thermodynamics), and the overpotential can be approximated by the potential at which the free energy change of all steps become negative (considering potential-dependence). Note that although this model gives the thermodynamic overpotential instead of the kinetic overpotential, it has been shown to yield semi-quantitative trend between the two that is consistent with experiment for various catalyst surfaces, comparable with more sophisticated models that explicitly calculate PCET free energy profile^45,63,64^.

### Simulated annealing sampling

Considering the complexity of the solvent environment, we combined AIMD simulation, simulated annealing (SA) method and static DFT calculations to explore the free energy surface and obtain chemically relevant configurations of the reaction intermediate, following the protocol we reported in ref. ^7^ For each pseudo-adsorption intermediate, we initially performed ~30 ps MD simulation to equilibrate the system. After that, five snapshots are randomly extracted from the equilibrated MD trajectory for each reaction intermediate as the initial configuration for SA. The SA is performed with a cooling rate of 0.9966 (the temperature in each step will be 0.9966 times that of the previous step) until 0 K to obtain the local minimum configuration. This approach allows the snapshot to have enough time to get relaxed and can relatively avoid the unreasonable or chemically irrelevant local-minimum structure. The most stable structure from the SA simulations was further chosen to be optimized and was considered as the intermediate configuration during ORR.

### Analysis of the AIMD trajectory

The radial distribution functions are calculated using the analysis model in VMD software version 1.9.4a48^65^. The coordination number of O by H CN_O_ is analytically calculated using an analytical expression adapted from ref. ^48^

1 $${\rm {C{N}}}_{{\rm {O}}}\left(R\right)={\sum }_{J=1}^{{N}_{{{{{{\rm{H}}}}}}}}\frac{1-{\left(\frac{\left|{R}_{I}-{R}_{J}\right|}{{r}_{0}}\right)}^{16}}{1-{\left(\frac{\left|{R}_{I}-{R}_{J}\right|}{{r}_{0}}\right)}^{32}}$$

Here ${r}_{0}$ = 1.32 Å. The local water density ${\rho }_{{{{{{{\rm{H}}}}}}}_{2}{{{{{\rm{O}}}}}}}$ as a function of *R* is calculated by effective counting along *R* within the region of 1.67 < CN_O_ < 3 for each subplot in Fig. 3b. The density distribution of the pseudo-adsorbed hydroxide species P(OH^δ−^) as a function of $R$ is obtained similarly based on effective counting along $R$ within the region of CN_O_ < 1.67 and $R$ > 2.5 Å (to exclude the *OH on Fe center) for each subplot in Fig. 3b.

### Calculation of work function

The work function is calculated based on the surface charge density-work function relation proposed in ref. ^66^

2 $$\phi =\frac{1}{C}\sigma +{\phi }_{{{{{{\rm{PZC}}}}}}}$$

The $\sigma$ is the surface charge density calculated from Bader analysis on equilibrated MD trajectory. The C and ${\phi }_{{{{{{\rm{PZC}}}}}}}$ are experimentally measured interfacial capacitance and potential of zero charge, respectively, taken as 21 μF/cm^2^ and −0.07 V in SHE scale from refs. ^67,68^

### Analytical models for solvation energy and electric potential energy

The solvation energy ${E}_{{{{{{\rm{solvation}}}}}}}$ that the water environment exerts on the hydroxide anion is based on the assumption that the solvation energy is proportional to the local water density distribution:

3 $${E}_{{{{{{\rm{solvation}}}}}}}\left(R\right)={E}_{{{{{{\rm{solvation}}}}}}}^{\ominus}\frac{1}{2{r}_{{{{{{\rm{solv}}}}}}}}{\int }_{R-{r}_{{{{{{\rm{solv}}}}}}}}^{R+{r}_{{{{{{\rm{solv}}}}}}}}\frac{\rho \left(r\right)}{{\rho }_{{{{{{\rm{bulk}}}}}}}}{{{{{{\rm{d}}}}}}r}$$

${E}_{{{{{{\rm{solvation}}}}}}}^{\ominus}$ is the hydration free energy of hydroxide, taken as −4.61 eV from ref. ^29^ The integration term is the averaged local water density divided by the bulk value, which describes the bulk-likeness of the local environment of the hydroxide. The ${r}_{{{{{{\rm{solv}}}}}}}$ is the hydration radius of the hydroxide species, here we approximate it by the thickness of a water bilayer, which is taken as ($1+\frac{\sqrt{2}}{4}$) times the O–O distance (2.72 Å) in a H-bond pair in liquid water, taken from the O–O RDF in our AIMD simulation. The electric potential energy contribution ${E}_{{{{{{\rm{coulomb}}}}}}}$ between the catalyst surface (positive charge localized on Fe center) and the hydroxide anion is approximated by the Coulomb’s law between two point charges:

4 $${E}_{{{{{{\rm{coulomb}}}}}}}\left(R\right)=k\left(R\right)\frac{{Qq}}{R}=\frac{1}{4\pi {\epsilon }_{0}\kappa \left(R\right)}\frac{{Qq}}{R}$$

Here $Q$ and $q$ are taken as +1 and −1 since Bader charge analysis suggest the positive and negative charge densities to be highly localized at the Fe center and the hydroxide anion, respectively. The relative permittivity $\kappa \left(R\right)$ is assumed to be proportional to the averaged water density between catalyst surface and the hydroxide:

5 $$\kappa \left(R\right)={\kappa }_{0}+\left({\kappa }_{{{{{{\rm{water}}}}}}}-{\kappa }_{0}\right)\frac{1}{R}{\int }_{0}^{R}\frac{\rho \left(r\right)}{{\rho }_{{{{{{\rm{bulk}}}}}}}}{{{{{{\rm{d}}}}}}r}$$

Here ${\kappa }_{{{{{{\rm{water}}}}}}}$ and ${\kappa }_{0}$ are dielectric constants of water and vacuum, taken as 78.4 (exp. value at 298.15 K) and 1, respectively. *ρ*_bulk_ is the water density of bulk water, taken to be 1.0 g/cm^3^.

## Supplementary information

Supporting Information

Dataset 1

## Data Availability

Coordinates and input parameters for setting our computational model and reproducing our findings are provided in the Supplementary Dataset. Additional data including the MD trajectories, and results of energy calculations are available from the corresponding author upon request.
