# Supplementary material for: Pseudo-adsorption and long-range redox coupling during oxygen reduction reaction on single atom electrocatalyst
Source: Nat Commun. 2022 Apr 1;13:1734. doi: 10.1038/s41467-022-29357-7 (PMC8975818; doi:10.1038/s41467-022-29357-7)
Supplement: Supplementary file 1 — Supporting Information [file 41467_2022_29357_MOESM1_ESM.pdf]

Supporting information

Pseudo-adsorption and Long-range Redox Coupling during Oxygen Reduction Reaction on Single Atom Electrocatalyst

Jie-Wei Chen[1,2#], Zisheng Zhang[1,2,3,+,#], Hui-Min Yan[1,2#], Guang-Jie Xia[1,2], Hao Cao[1,2], Yang-Gang Wang[1,2,*]

[1]Department of Chemistry, Southern University of Science and Technology, Shenzhen 518055, Guangdong, China

[2]Guangdong Provincial Key Laboratory of Catalysis, Southern University of Science and Technology, Shenzhen 518055, Guangdong, China

[3]Department of Chemistry and Biochemistry, University of California, Los Angeles, 607 Charles E. Young Drive East, Los Angeles, California 90095, United States

Email: wangyg@sustech.edu.cn

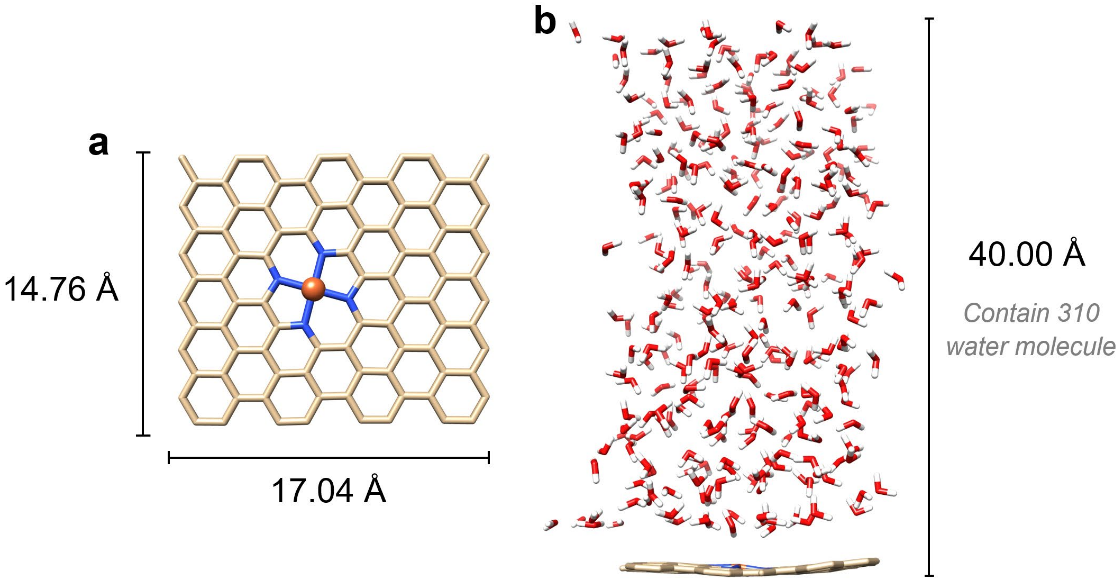

Supplementary Figure 1. Computational models. (**a**) The Fe-N_4/C catalyst modeled by a layer of graphene with Fe-N_4 embedded in the center. (**b**) The Fe-N_4/C catalyst with solvent environment used in MD simulation.

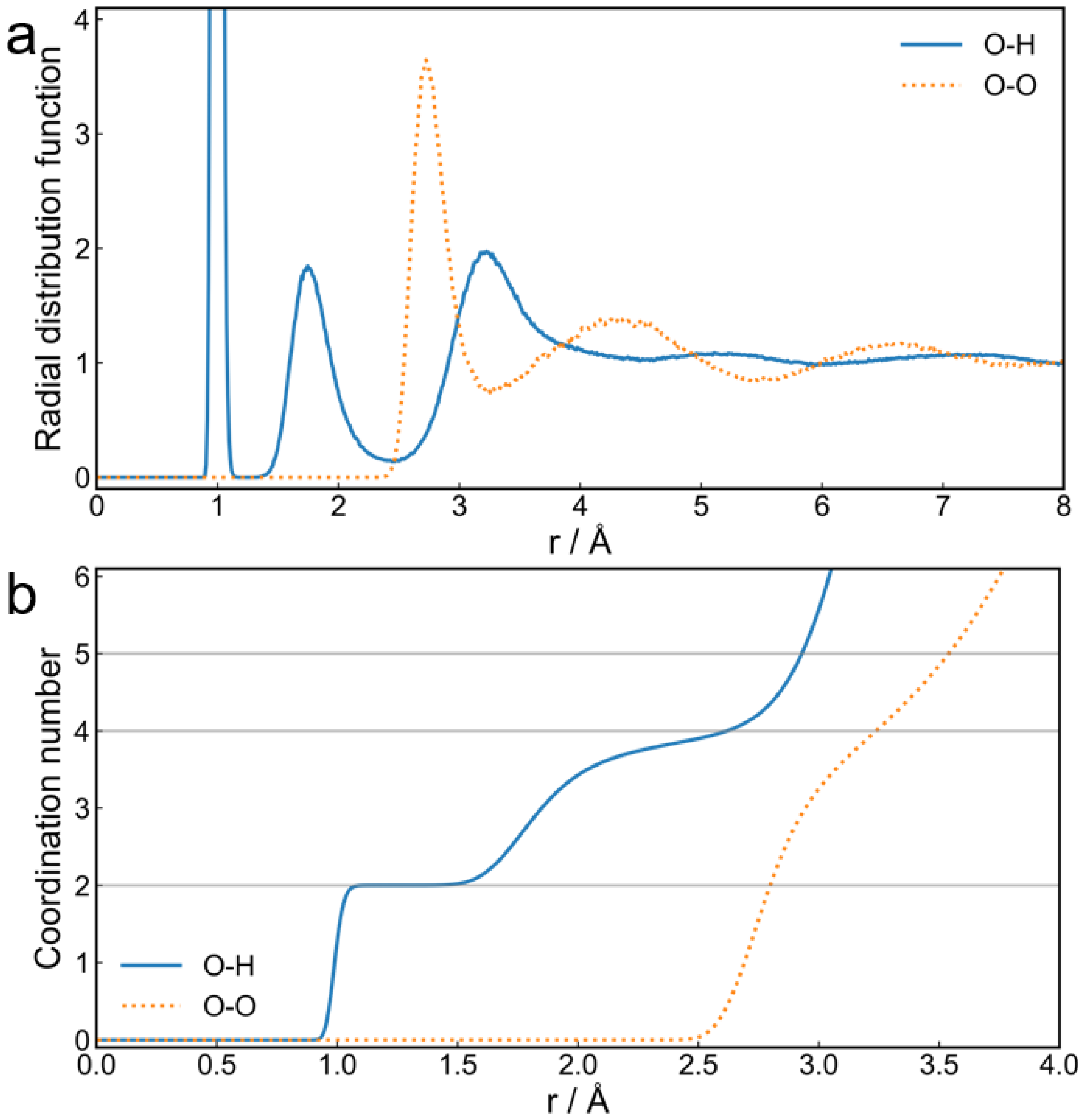

Supplementary Figure 2. Radial distribution function of water from equilibrated AIMD trajectory. (a) The radial distribution function (RDF) of O-H and O-O and **(b)** the integrated RDF of O-H and O-O showing the coordination numbers.

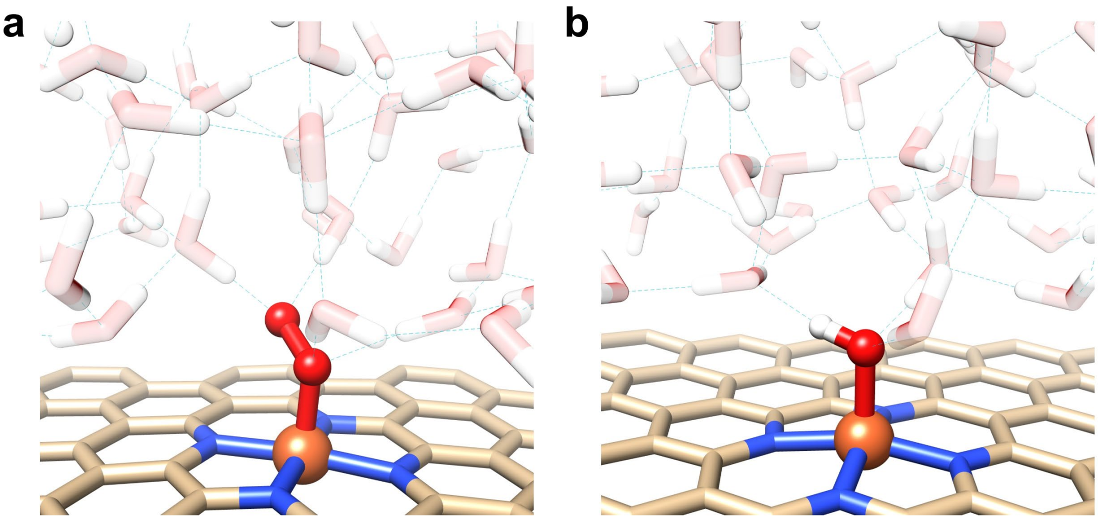

Supplementary Figure 3. MD Snapshots of some reaction intermediates. (a) The *O_2 species possess a side-on configuration and forms about 3 hydrogen bonds with the surrounding water molecules. **(b)** The *OH species forms about 2 hydrogen bonds with the surrounding water molecules.

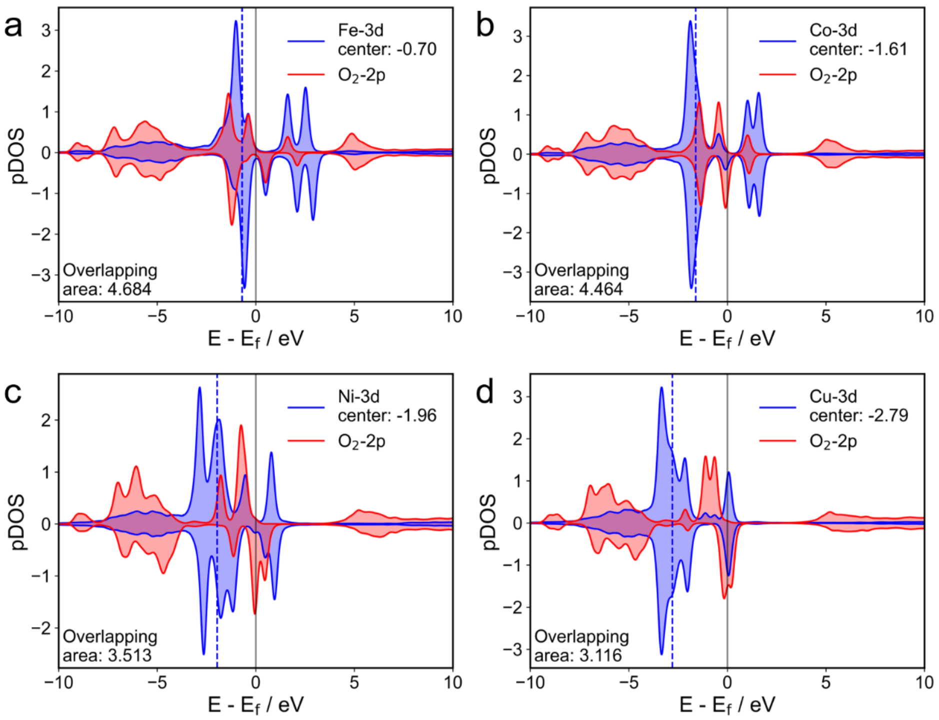

Supplementary Figure 4. The projected density of states (pDOS) The pDOS of O 2p and 3d states of **(a)** Fe, **(b)** Co, **(c)** Ni, and **(d)** Cu upon O2 adsorption for the GP model. Grey solid line and blue dashed line represent the fermi level and the d band center of the metal atoms. The d band center value is given in the legend, and the integrated overlapping area is labeled in the lower left.

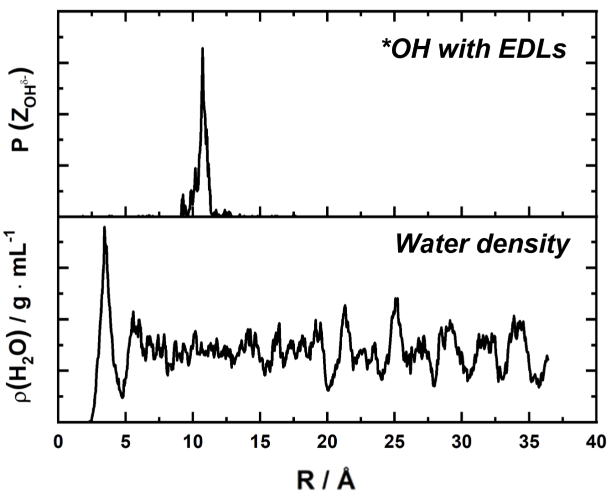

Supplementary Figure 5. The exploration for pseudo-adsorption state of $OH^{\delta-}$ in *…$OH^{\delta-}$ with EDLs. The probability distribution functions P ($OH^{\delta-}$) of pseudo-adsorbed $OH^{\delta-}$ (upper panel) and the water density distribution (lower panel) of the system *…$OH^{\delta-}$ with EDLs.

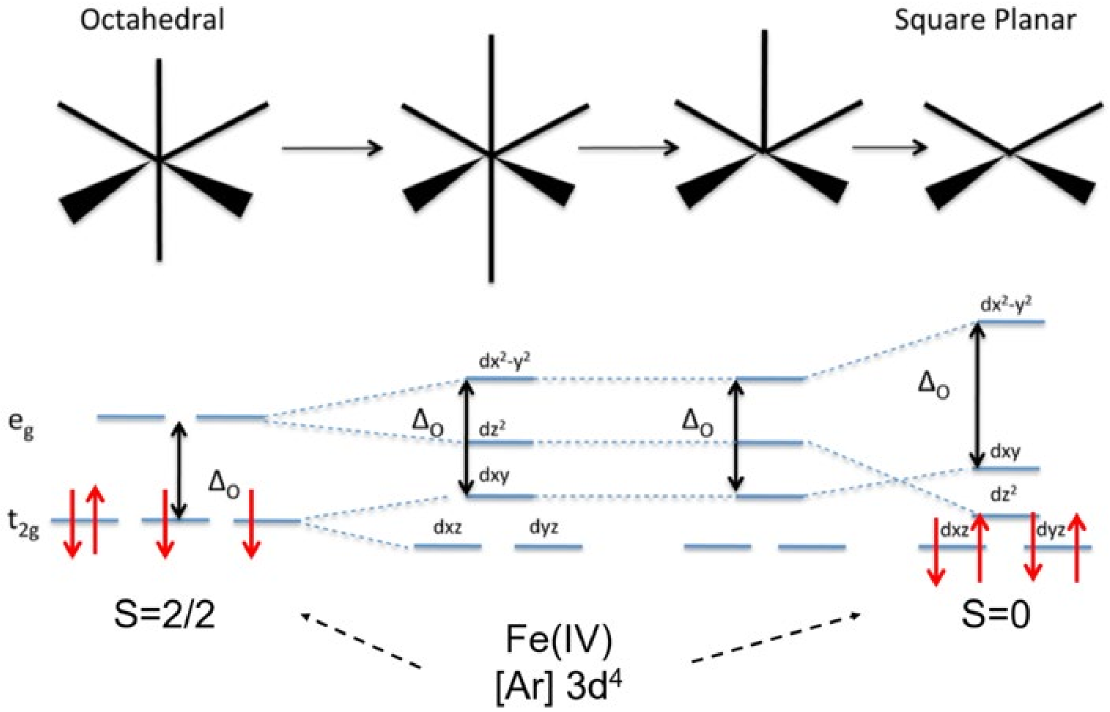

Supplementary Figure 6. The spin state of Fe(IV) in different crystal fields. The red arrows represent 3d electrons of Fe(IV), and the resulting total spin S are labeled below the octahedral and square planar orbitals, respectively.

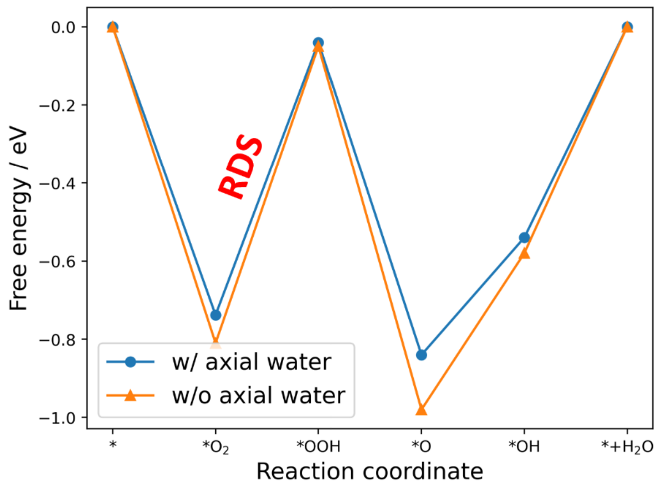

Supplementary Figure 7. Free energy diagram for oxygen reduction reaction over Fe-N_4/C single atom catalyst in gas phase with or without backside axial water ligand, at 1.23 V_{SHE}.

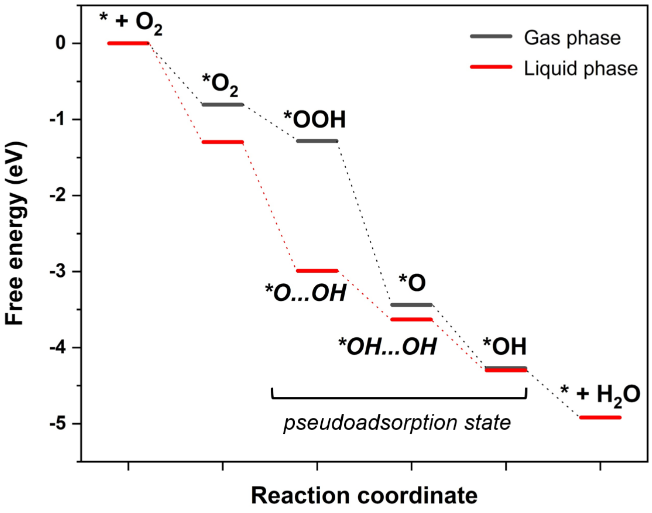

Supplementary Figure 8. Free energy diagram for oxygen reduction reaction over Fe-N_4/C single atom catalyst in both gas and liquid phase at 0 V_{SHE}.

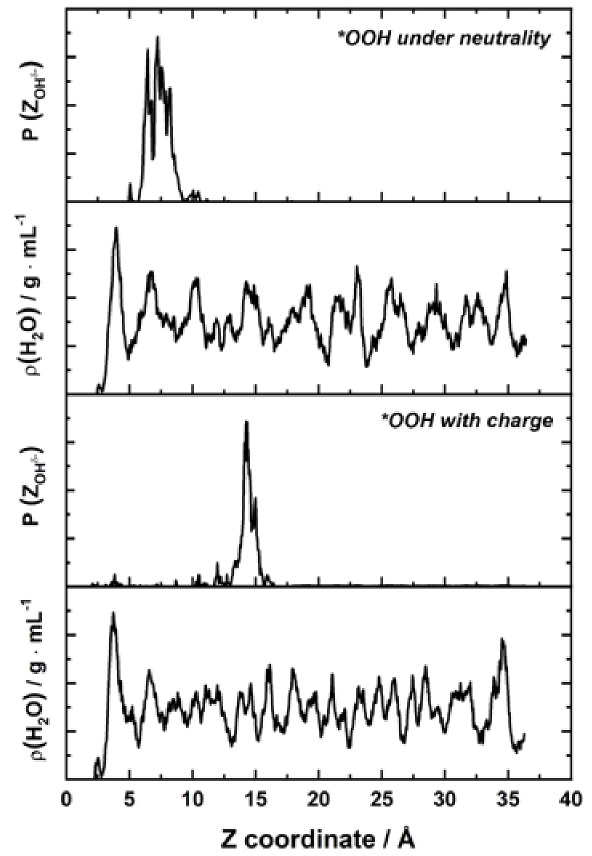

Supplementary Figure 9. The exploration for pseudo-adsorption state of $OH^{\delta-}$ in *…$OH^{\delta-}$ with extra charge in the surface. The probability distribution functions P ($OH^{\delta-}$) of pseudo-adsorbed $OH^{\delta-}$ (upper panel) and the water density distribution (lower panel) of the system *…$OH^{\delta-}$ with EDLs.

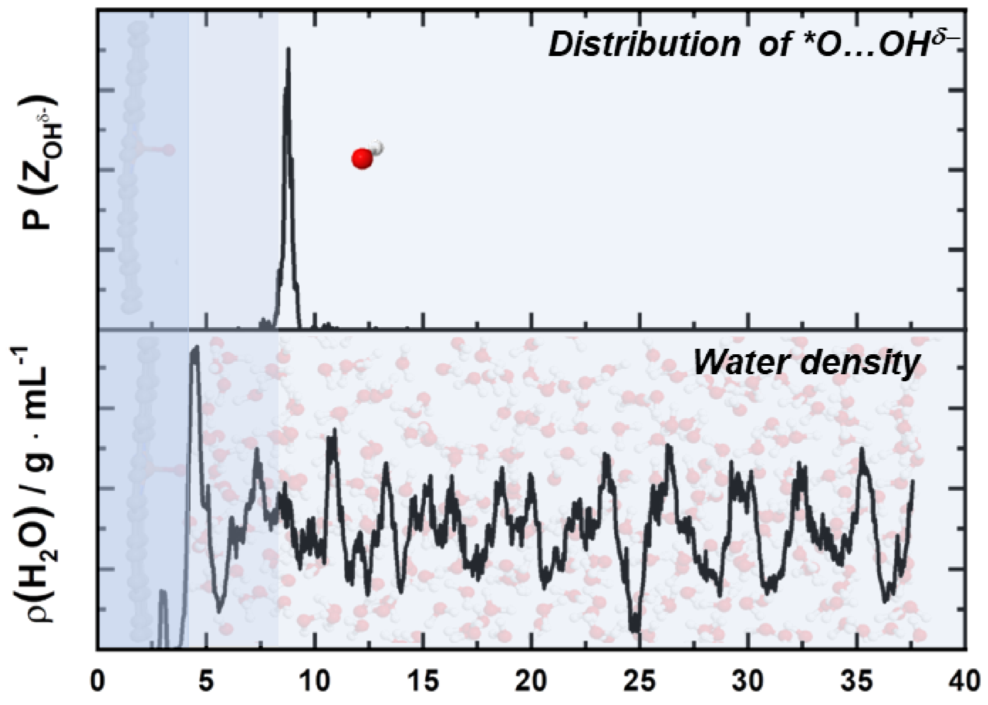

Supplementary Figure 10. The exploration for pseudoadsorption state of $OH^{\delta-}$ in *O…OH reaction intermediates reproduced by SCAN meta-GGA functional. (a) Thermodynamic insight into the pseudoadsorption state. **(b)** The probability distribution functions P ($OH^{\delta-}$) of pseudoadsorbed $OH^{\delta-}$ and water density of *O…$OH^{\delta-}$.

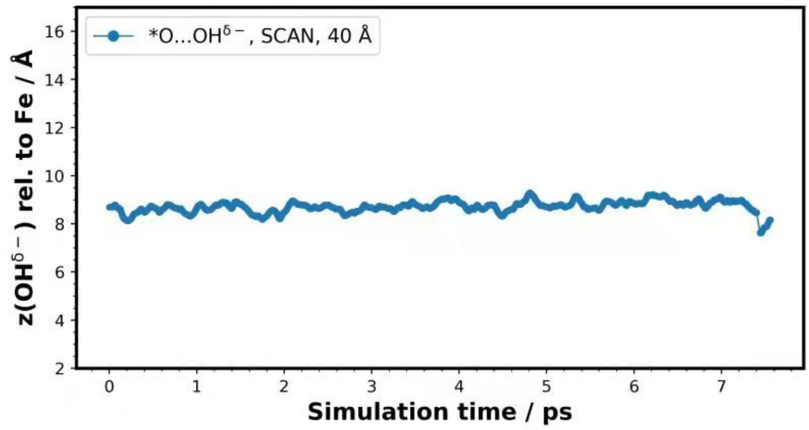

Supplementary Figure 11. Spatial evolution of the pseudo-adsorption species from AIMD reproduced by SCAN meta-GGA functional. The z coordinate of the O in dissociated $OH^{\delta-}$ relative to that of the Fe single site in *O…$OH^{\delta-}$ intermediates during the AIMD simulation.

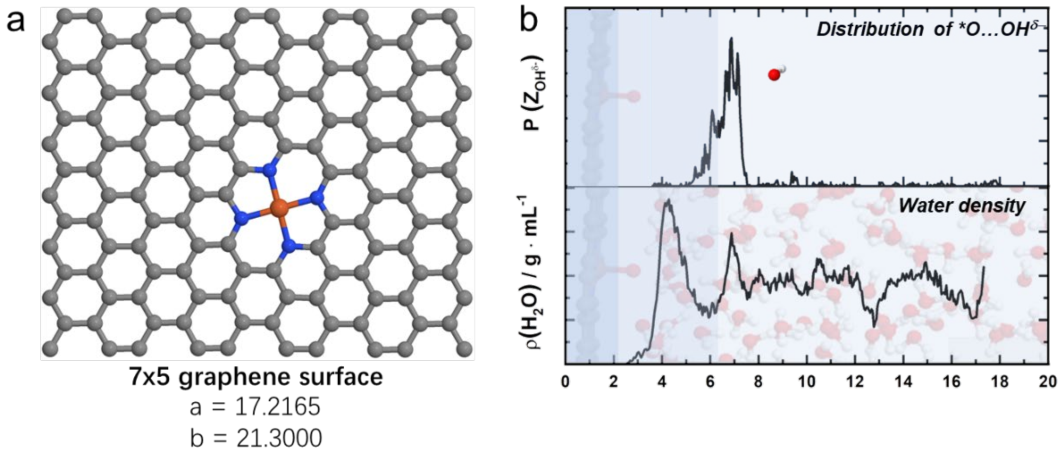

Supplementary Figure 12. The exploration for pseudoadsorption state of $OH^{\delta-}$ in *O…OH reaction intermediates reproduced by larger size of surface unit cell. (a) The FeN4/C catalyst model composed by 7 × 5 graphene supercell. **(b)** The probability distribution functions P ($OH^{\delta-}$) of pseudoadsorbed $OH^{\delta-}$ and water density of *O…$OH^{\delta-}$.

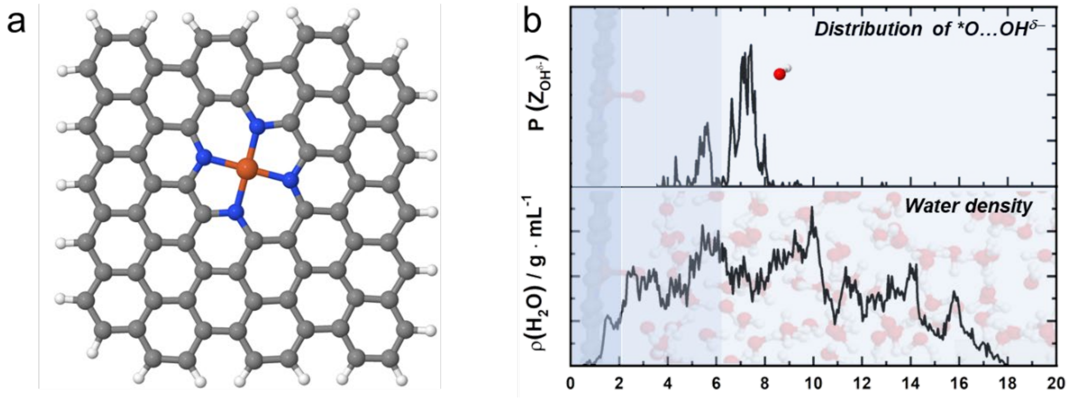

Supplementary Figure 13. The exploration for pseudoadsorption state of OH$^{\delta-}$ in *O…OH reaction intermediates reproduced in non-periodic system. **(a)** The FeN4/C catalyst model for non-periodic system calculation. **(b)** The probability distribution functions P (OH$^{\delta-}$) of pseudoadsorbed OH$^{\delta-}$ and water density of *O…OH$^{\delta-}$ in non-periodic system.

Supplementary Table 1. The surface charge density and SHE-scale workfunction of the reaction intermediates.

Reaction intermediate	σ_{surf} (C/cm^{-2})	ϕ_{SHE} (V)
$*O...OH^{\delta-}$	11.25	0.47
$*OH…OH^{\delta-}$	9.11	0.36
$*…OH^{\delta-}$	4.20	0.13
$*O...OH^{\delta-}$ (1 extra electron)	6.80	0.25
$*O...OH^{\delta-}$ (w/ Na^+ counter ion)	6.77	0.25

Supplementary Table 2. The Mulliken spin density and the Fe Bader charge within the relative OOR reaction intermediates.

Reaction intermediate	Fe Bader Charge	Mulliken spin population						
		N1	N2	N3	N4	N total	Fe	Fe-N4 total
* (FeN_4)	1.183	0.000	0.000	0.000	0.000	0.000	0.000	0.000
*O_2	1.257	-0.021	-0.020	-0.027	-0.017	-0.086	0.560	0.474
*O...$OH^{\delta-}$	1.338	-0.041	-0.034	-0.044	-0.033	-0.152	1.290	1.138
*OH…$OH^{\delta-}$	1.286	-0.014	-0.033	-0.022	-0.027	-0.096	0.670	0.574
*…$OH^{\delta-}$	1.200	0.009	0.003	-0.001	0.012	0.023	0.280	0.303

Supplementary Table 3. The spin state and formal oxidation state of Fe center predicted by the proposed mechanism in this work.

Reaction intermediate	Fe formal O.S.	# Unpaired e^- estimated from Square Planar Crystal Field	# Unpaired e^- estimated from Octahedral Crystal Field
* (FeN_4)	2	0	0
*O_2	3	1	1
*O...$OH^{\delta-}$	4	0	2
*OH…$OH^{\delta-}$	3	1	1
*…$OH^{\delta-}$	3	1	1

Supplementary Table 4. The Gibbs free energy differences for the relative reduction steps of ORR in liquid phase.

Reaction step	ΔG (eV)	Accumulated ΔG (eV)
$O_2 + * = *O_2$	-1.30	-1.30
$*O_2 + H^+ + e^- = *O...OH^{\delta-}$	-1.69	-2.99
$*O...OH^{\delta-} + H^+ + e^- = *OH…OH^{\delta-}$	-0.64	-3.63
$*OH…OH^{\delta-} + H^+ + e^- = *OH + H_2O$	-0.67	-4.30
$*OH + H^+ + e^- = * + H_2O$	-0.62	-4.92

Supplementary Table 5. The Gibbs free energy differences for the relative reduction steps of ORR in gas phase.

Reaction step	ΔG (eV)	Accumulated ΔG (eV)
$O_2 + * = *O_2$	-0.81	-0.81
$*O_2 + H^+ + e^- = *OOH$	-0.48	-1.28
$*OOH + H^+ + e^- = *O + H_2O$	-2.16	-3.44
$*O + H^+ + e^- = *OH$	-0.83	-4.27
$*OH + H^+ + e^- = * + H_2O$	-0.65	-4.92

Supplementary Table 6. The Gibbs free energy differences (in eV) for generation of the pseudo-adsorbed hydroxides in the first PCET step in the proposed ORR mechanism in liquid phase.

Reaction step\Functional	PBE	SCAN	PBE0	HSE06
$*O_2 + H^+ + e^- = *O...OH^{\delta-}$	-1.40	-2.41	-1.05	-0.66
$*O...OH^{\delta-} + H_2O = *OH...2OH^{\delta-}$	0.33	0.33	0.15	0.18
$*O...2OH^{\delta-} + H_2O = *OH...3OH^{\delta-}$	0.59	0.80	0.26	0.17
