# Supplementary material for: Pseudo-adsorption and long-range redox coupling during oxygen reduction reaction on single atom electrocatalyst
Source: Nat Commun. 2022 Apr 1;13:1734. doi: 10.1038/s41467-022-29357-7 (PMC8975818; doi:10.1038/s41467-022-29357-7)
Supplement: Supplementary file 2 — Dataset 1 [file 41467_2022_29357_MOESM2_ESM.pdf]

Supplementary Dataset

Coordinates and input parameters for setting our computational model and reproducing our findings

1. Initial structure for AIMD simulation of *OOH on FeN_4/C catalyst

C	11.5018864854	7.8026513878	0.0485267686
C	12.2071630878	9.0293924751	0.0576209657
C	11.5049159251	10.2551216364	-0.0051201486
C	10.0828647151	10.2549803841	-0.0565513360
C	11.4722197931	12.7128847349	-0.0061935332
C	10.1015126716	7.8006145683	0.0053523597
C	10.0494160832	12.7095628458	-0.0575318408
C	12.1911783027	11.4837236800	0.0338664105
C	9.3876682616	9.0395732599	-0.0409518398
C	12.1785552917	6.5602699205	0.1256289366
C	9.3601888470	11.4751226117	-0.0611460576
C	13.6157313024	9.0160986379	0.1603537337
C	12.1631389414	-0.8177080877	0.0706587541
C	11.4347514308	5.3560587307	0.0963404660
C	11.4475768651	0.4159195085	0.0568690704
C	10.0227548848	0.4214525466	0.0114627964
C	9.3336407962	-0.8202521345	-0.0197059442
C	13.5945335205	6.5504544371	0.2224329294
C	13.6114634543	11.4739495851	0.1506146232
C	12.1337446824	4.1091202298	0.1302263017
C	14.3086028296	7.7772636014	0.2405605463
C	11.4243049353	2.8903641606	0.0869756215
C	14.3179499354	10.2420974395	0.2007322705
C	12.1374872709	1.6500369557	0.1204029777
C	13.5620417935	4.0904369095	0.2296366452
C	10.0034126838	5.4151703113	0.0531871472
C	13.5916899662	-0.8290482541	0.1842607524
C	14.2869531850	5.3094454360	0.2799548736
C	9.9860490877	2.9223334088	0.0435334449
C	7.9382753184	11.4191733278	-0.0055872027
N	8.0195373222	8.9772103974	-0.0312117257
C	9.3075986170	1.6620949961	0.0368127703
C	14.3094120620	12.7006534054	0.2278053084
N	9.3729615667	6.6472335710	0.0499359029
C	13.5637470151	1.6315684410	0.2253501199
C	14.2684884222	2.8521549632	0.2898824781
C	7.9116873256	13.9070696740	0.0540988521

C	15.7257990735	7.7677342774	0.3093485293
C	14.2841882674	0.3954027089	0.2637448514
C	9.2663552909	4.1760660659	0.0373295309
C	15.7401487468	10.2299624433	0.2855264059
C	7.2814662646	10.1473652131	0.0110962061
C	15.7078101751	5.2992083606	0.3558972514
C	7.2189461655	12.6533847849	0.0683533080
C	-0.6038294245	8.9942646582	0.3098619381
C	15.7318825314	12.6839175063	0.3208674638
C	16.4218803170	6.5261245379	0.3533270933
C	15.7001274139	2.8376648959	0.3897668414
C	7.8909328656	1.6360692963	0.0821830609
C	-0.5983029394	11.4504175197	0.3225595276
C	15.7117170265	0.3778903008	0.3637542447
C	16.4067059692	4.0610424334	0.4023539701
C	7.2013432478	0.3818610689	0.1334098690
C	7.8234214499	4.1427893898	0.0201940603
C	-0.6113024588	13.9055712702	0.3730261418
C	16.4141178246	1.6025026577	0.4211396763
C	0.8156479606	8.9810177032	0.3026505546
C	0.7964565196	6.5161375193	0.3487876310
C	5.8405277806	10.1140971370	0.0891171386
C	7.1690926166	2.8706289871	0.0769303847
C	0.8322174092	11.4344292773	0.3266731229
C	5.8057011412	12.6264793839	0.1674881243
C	1.5081560368	7.7410170372	0.2965155446
C	1.5390871987	10.1962712386	0.2827735717
C	0.7885804120	4.0492835106	0.4036214592
N	7.0796349305	5.3125529709	-0.0226341411
C	5.1249907355	11.3660425481	0.1623455706
C	1.4895038548	5.2767560073	0.3482183069
C	2.9739969278	10.1791268292	0.2236896245
C	0.8218007282	13.8896403553	0.3941830311
C	3.6727054552	8.9325673555	0.1639881445
C	5.1048456831	8.8732813205	0.0972533104
C	0.7977410754	1.5866334610	0.4371197006
C	1.5409222820	12.6562414552	0.3506768896
C	2.9218333281	7.7283248854	0.2092442182
C	3.6881085045	11.3975952497	0.2335481202
C	1.4954082393	2.8142909175	0.4081992820
C	5.7826768823	0.3485794672	0.2375433155
C	5.0945281870	13.8662875329	0.2570749244
C	1.5164718643	0.3552312938	0.4123672320
C	2.9722461815	12.6378363256	0.3107289040

N	5.7341045929	7.6418027767	0.0634782055
C	5.7107859211	5.2488575819	0.0350711269
C	3.6002265984	6.4856558792	0.1648645738
C	5.7470482808	2.8144522330	0.1466743986
C	2.8985876997	5.2605722140	0.2534976524
C	5.0000143358	6.4876676577	0.0652186103
C	3.6660032748	13.8715297125	0.3229709276
C	5.0208497607	4.0327403560	0.1285867836
C	2.9202127293	2.8056346358	0.3346826749
C	2.9472722047	0.3447912895	0.3693752495
C	5.0648043813	1.5801764769	0.2489392232
C	3.6030186603	4.0339104948	0.2403511439
C	3.6416270659	1.5771629934	0.3259482422
Fe	7.5534323432	7.1432813480	0.2404050482
O	7.7073490274	7.0794321793	2.0361127602
H	13.8722945832	5.3684257227	2.6871537309
H	4.2713530350	12.2923141647	3.2408119528
H	-2.9137846216	13.1807141957	2.6452895938
O	9.9802209715	-1.8619768496	2.9859386180
H	10.3959910731	-0.9710127998	3.0905525810
H	10.6251978023	-2.5414237005	3.2947159673
H	8.5048204943	5.6088754927	2.8379775437
O	11.4948306443	3.5797325177	3.3393759883
H	-0.6141529895	8.5758892506	3.2653655752
H	11.3816560929	2.5931671299	3.2487142391
H	8.3412225895	12.8917813068	3.2573465382
O	6.4993907093	7.3581055403	2.8068826371
H	-3.6392000138	9.8580121028	3.5522065175
H	10.6416551499	3.9828557111	3.0819512924
O	10.8718251269	0.8493521553	3.4155313124
O	-2.7489606573	9.4046917901	3.5843480210
H	6.1277053445	6.4400442313	2.8986179671
H	12.8391792008	4.4858525802	3.4859380559
O	4.5633914265	2.4575148179	3.4886876979
H	11.2040301272	9.8598681036	3.7185830169
H	3.9911611155	2.0988552872	2.7837676510
H	0.9529476964	8.6548181701	3.4961043954
H	-1.4347208623	14.8216558274	3.6077670124
O	13.6825265888	5.0414033274	3.5875614055
O	-2.5854268451	13.4679785779	3.5179458162
H	5.8161597254	12.0416280077	3.3105330926
O	11.7865688003	10.6714065695	3.8277133196
O	7.3832696977	12.8865101698	3.5825145277
O	10.2255651013	8.4467579366	3.6606580946

H	5.2232518610	1.7183030323	3.7128470183
O	4.9310100975	11.6208390194	3.4885234204
O	0.1293869270	8.1484610331	3.7289456024
H	7.1168546240	13.8325010463	3.7335002614
H	3.0882078563	9.5753347785	2.7367875492
H	1.0029053218	0.2841335747	3.8285941196
H	10.5652523766	7.6038004119	4.0311368450
H	10.0482751735	1.1697973700	3.8585004137
H	1.8556126296	-0.9998833912	3.8802201577
H	-1.0854073577	16.3410510395	4.0139012673
O	-0.7523836401	15.4060084579	4.0317508058
O	1.9299621816	-0.0240040421	4.0005557620
O	2.4771817739	9.5360537806	3.4958514277
H	-2.0972398890	3.9162862039	4.0978964214
H	-2.1247505550	12.6654235400	3.9181199495
H	-2.8768886225	8.6274194918	4.1884599618
O	8.7222621894	4.8758994538	3.4745019119
H	1.8314603092	11.0983608995	3.8787448861
O	6.4121063240	0.6877249805	4.2308234284
H	7.2301162924	1.2601635392	4.3330148751
H	15.2935435634	10.5615676213	4.1691627216
O	1.3297746406	11.9365291335	4.0863446144
H	11.4790378389	0.5291166027	4.1590383312
H	8.6544965447	3.0574752293	4.0730609702
H	16.6804207103	11.4585636555	4.2276889935
O	-1.5406846470	3.2171544244	4.5405077396
H	9.4160919646	8.6160680012	4.1997850131
H	9.2394272428	5.3323940580	4.2135321301
O	8.6414360564	2.1839423319	4.5276637838
H	5.5003233143	3.9049251572	3.3154973261
H	11.8026382683	10.8040079400	4.8066806758
O	15.7526198007	11.3600455122	4.5939024137
H	13.2421386599	-0.5565372387	4.6418619497
H	13.7902439400	6.4130527261	4.7684459506
H	-0.7636642542	3.6723692356	4.9691878014
H	3.6529247709	3.0813610630	4.8903604102
H	6.9272528901	4.6079569066	3.6346270088
H	3.0164669942	9.1403145874	4.2651356467
H	6.1078543081	0.5299548007	5.1737334068
H	7.6745891683	12.1380674702	5.0309025562
H	1.4042593661	12.0798955097	5.0713320282
O	10.4010317144	5.9548492431	5.2204325552
H	16.9825191008	7.4724195338	5.2436574181
O	14.0846235906	7.1966838054	5.3077229077

O	5.9538893593	4.7710721600	3.5190456723
O	12.5823955878	-0.1072002015	5.2266984467
H	15.0231395777	6.9907135944	5.5976421817
H	11.0117682357	5.2313425734	5.5259610841
H	8.9529202697	2.3634705325	5.4654598493
H	4.9780672432	11.1759611038	5.1076206901
H	14.4086088478	2.5687947922	5.6770875847
H	18.4833725589	4.1148880164	5.7603731497
H	13.0434975400	0.6970830235	5.5844374637
H	-0.8968746630	-0.0253963365	5.6360997010
O	3.2683783367	3.3900524032	5.7582534616
H	2.0279820650	0.6102675409	5.6430041427
O	17.5536296506	4.3851141500	5.9584160499
H	17.0785639264	6.0399217063	5.9566750355
O	16.6846890675	6.9554176689	6.0570734518
H	10.1990027161	6.4716360052	6.0485441629
O	8.3284913503	8.8855617617	5.8171840637
O	7.7612932683	11.6740522509	5.9273384906
H	2.8029320038	2.5391642065	6.1031520116
O	13.6918255353	2.1925172047	6.2700567499
H	5.1916810209	5.4997598839	4.7881969146
H	8.1757225753	9.8582836651	5.9135510157
H	-1.0702416886	10.9562621888	6.2157178970
H	12.5200537698	3.3769895472	6.4593341629
H	6.0919730631	6.5722691384	6.4563824620
O	12.0424046048	10.8013183090	6.6962143847
O	3.7468148648	8.5752842424	5.5738620784
H	6.1605326883	11.1872769920	6.1290404687
O	5.1815256139	10.9491731144	6.0723164355
H	4.0627302541	5.3045668263	5.8576611424
H	13.2797885958	7.9254491553	6.5884696166
O	2.0124355490	1.2322703813	6.4261887405
O	11.7970980035	4.0557602764	6.6356832484
H	4.1405241440	7.6728947865	5.4749315910
O	-0.9813254084	-0.5428035552	6.4901519238
H	8.0405464914	12.3946581771	6.5374424964
H	4.6009506768	3.2635325701	6.8465624934
H	9.0768838757	8.6534210970	6.4371458987
H	12.7073606639	11.5234731341	6.8897279712
H	17.3458263325	4.0231987673	6.8732279631
O	4.7310830987	5.9580101435	5.5608784799
O	5.6421191209	15.1304937484	6.8039747473
H	7.3250144800	7.6184612396	6.4531071264
H	0.5041636821	13.3254499039	6.6767104851

H	4.3505962642	12.2679736135	6.7822376307
O	1.2175543094	12.6177943747	6.7360035615
H	12.6146324180	9.3221713869	7.0729363496
H	10.4107920888	3.2393216335	6.9279992845
H	4.8682970524	14.5082113384	6.9154765844
H	14.4070710651	-1.5142991869	6.9345320204
H	14.1572464364	1.9007281056	7.1047589125
H	5.3999021958	16.0215419543	7.1759950973
H	2.9331638307	12.9546560830	7.0692288437
O	9.5259135620	2.7546543845	7.0390578717
H	11.3117615682	10.9215243896	7.3606613162
H	4.4740213277	9.2043172614	5.7909075467
O	6.9006897249	6.8615182190	6.9619807644
O	3.9146820966	13.0661072926	7.2040035691
H	-1.1539114727	0.1304867082	7.1942436485
H	0.7194911860	11.8728368162	7.1502616629
O	12.8491408627	8.3805547564	7.3785161899
O	-0.9148254807	10.8490662049	7.2106188474
H	12.1329328430	4.6212565709	7.3825925764
H	1.9639930873	0.7178707168	7.2697002661
H	6.0618985941	3.5241020759	7.5496096175
O	5.2105532930	2.9951039882	7.5980563534
H	-0.9000843039	9.8910130301	7.3824049849
O	10.1657330329	7.6662056925	7.3246729942
H	11.1122803776	7.9630266325	7.3263389261
H	7.0980473964	-0.2791131590	7.4487519969
O	13.9566550747	-2.1742086133	7.5194022584
H	7.5503018135	5.2607881415	7.4426108755
H	8.3236048643	3.8291676672	7.5370447038
H	-0.2474562884	7.2918620233	7.7332919561
H	14.6798606429	-2.8359481641	7.6708136303
H	9.7165681625	2.0074074001	7.6713286142
O	7.9082289638	-0.8042104920	7.7064774362
O	7.5406773621	4.3655245666	7.8782960743
H	5.0985294017	10.2521563210	8.0094047967
H	12.5742290752	6.6359373286	8.3760714599
O	15.2075171684	1.3273755308	8.3713887218
H	15.8444270621	2.1085360197	8.3734499457
O	-0.2837921849	3.5023792616	8.3862529310
H	8.5186050532	-0.2023112187	8.2022973220
H	9.9327514193	7.5412661193	8.2999159848
O	12.6908750814	5.6982140401	8.6402531077
H	6.5181752204	7.3390987557	8.5069071482
H	12.5206437382	-1.5064133670	8.5844105044

O	-0.4409761529	7.4154496747	8.7103469103
H	13.8897643678	8.7645487271	8.7565951079
O	10.2861754506	11.1352868725	8.8401322544
H	-0.8867442739	4.1546492537	8.8515120295
H	7.5652876156	12.6144651882	8.6337892715
H	9.2986370413	11.2912761741	8.8006625547
H	4.4850017791	3.3972114348	8.9898033662
H	4.2145945618	-1.4017795729	8.8831083832
H	0.4358010498	3.2581435540	9.0476471338
H	11.1795022823	-2.1727307663	9.0823725943
O	5.3289556559	10.1784792171	8.9602453041
H	14.2578026389	5.4294920513	9.2100538778
O	1.7499535467	0.1166371762	9.0072830855
O	9.9108974685	0.7863169264	8.9831479272
H	10.6574122116	0.1375307138	9.0525565037
H	5.7140389093	9.2580385918	9.0783017000
H	0.4047137439	7.4991011504	9.2383123887
H	15.3476085989	8.3691791200	9.2041046168
H	14.8335105719	1.3238796636	9.2969178529
O	11.8018133984	-1.4032746691	9.2564092378
O	7.6914835733	11.7846025643	9.1843825516
H	6.8515312400	11.2571802724	9.1592281288
H	15.6770694011	6.0761415579	9.4404770749
H	2.6199827463	-0.2309525635	9.3636118999
H	12.1232544331	5.5415034887	9.4637575850
O	6.2756770746	7.6860578928	9.4256263317
H	5.5287582379	7.1380922010	9.7220446925
H	1.6248473261	0.9949032834	9.4735514054
O	14.5452132988	8.8978390419	9.4992471058
O	15.1337444130	5.2749358597	9.6757531712
H	0.4947759091	13.9170425527	9.4950483090
H	10.3770023090	10.6040264597	9.6816161586
H	7.7442984130	4.4652299323	9.5927138425
O	4.0509972358	3.4575587439	9.9052330036
O	4.0638216291	-1.0325485344	9.7966397968
H	2.4978685955	2.8815527613	9.9167972071
H	-1.0538227671	14.0720066679	9.6537408420
H	9.9535573312	1.3451727014	9.8037717969
O	9.6711856588	7.3766217153	9.9137252720
H	3.8366542261	9.8663314410	9.9751683164
O	1.5495489153	2.5802585530	10.0613280184
O	-0.3247433797	13.4719378072	9.9015406859
H	3.8589153655	4.3944214969	10.1572979049
H	4.9094558516	-0.6254420667	10.1470363857

H	15.1637013499	10.4184096805	10.0699315167
H	16.0208440804	11.7932966327	10.1531153935
H	10.1402282481	6.5931629100	10.2965871188
H	2.0144513604	8.4271699887	10.5773571768
H	9.9611942186	8.1698694281	10.4412502920
O	1.4870633869	7.5693925269	10.5906706713
O	3.1075444987	9.7148919970	10.6356350730
H	12.6585185785	12.7352724707	10.6158098741
O	7.8554009213	4.3173512553	10.5786961130
H	8.2119031800	-2.1203690636	10.6355361784
O	15.4376414952	11.1749940087	10.6611017110
H	5.2298368346	2.8946606655	10.9858687669
O	11.2541460857	5.2199333688	10.8023618004
H	2.8913269978	10.6557934794	10.9819487486
H	3.4257546242	12.6730535378	10.9579765174
O	14.5636348248	1.4428125670	11.0129105305
H	9.1828491116	3.2854358869	10.8988540623
H	1.4163086621	2.6867730361	11.0531370485
H	7.7651647875	7.2649726784	11.0562165270
H	2.5162981221	6.3243418487	11.0665045421
H	14.0524819920	12.0340350982	11.0457773652
H	7.8679127014	5.2057091426	11.0373749832
H	10.9163023831	4.2876421993	10.9474019231
O	6.1299431077	-0.0340897987	11.1575969126
O	10.0193067890	2.7619559376	11.0625679411
H	14.1781072217	8.4082867789	11.1617601467
H	6.7342332868	3.2740174472	11.2180497996
H	14.5817386730	4.6264479031	11.3090716584
H	14.4958262261	2.4024323747	11.2771897626
O	10.2154337299	9.7363468982	11.2183466916
H	6.2528890215	0.9506618439	11.2476891944
H	7.0210218474	-0.4975510150	11.2478845692
H	0.8446524959	7.6241623016	11.3610024831
O	3.1308593427	5.5812074162	11.3760792979
O	13.2058855077	12.4390987638	11.4057984607
H	13.8158575972	0.9750984972	11.5065375272
O	6.0189904352	2.6520845186	11.5537283606
O	8.2143766901	-1.6062532929	11.4849020275
O	2.9618178084	12.0887457191	11.6234244179
H	17.2420654398	-1.3236107521	11.5897758982
H	11.6450780847	5.5614227722	11.6576846705
H	9.3956611213	10.0202055691	11.7318945722
H	9.1485321926	-1.3740295484	11.7800890491
H	2.5557220960	4.9661655531	11.8721831811

H	10.9744643980	9.9202316102	11.8270748326
O	7.7418208397	6.7439804142	11.8909501167
H	2.2072373897	12.6365559883	11.9466556431
O	14.6206670617	3.9681012601	12.0523263492
H	3.6849273734	9.2106037299	12.1350742721
H	9.9848731641	2.5356240539	12.0310837299
H	15.7951761619	1.1937410204	12.1736085219
O	14.1022494523	8.2636554848	12.1472321971
H	13.1688382781	13.8976953426	12.2633054653
H	6.7941534602	6.7850594961	12.2197020741
H	4.5400338395	6.1761218526	12.3996222183
H	15.0316290642	8.0564794837	12.4387666689
H	12.6532303570	11.2263946844	12.4030414175
O	17.7827918796	-1.1105319338	12.4004197679
H	8.1737701759	11.6430512437	12.4037555550
H	15.8484547428	10.6377708664	12.4097395410
H	13.7860635176	3.9482671939	12.5831978669
O	12.8975715819	14.8121279122	12.5697044238
H	5.4019962577	14.1723383057	12.5902097762
H	12.8935412523	7.1702015883	12.6677446310
O	1.3182576461	2.8958959678	12.7180460726
O	16.7890388276	7.4996986766	12.7285832434
O	8.1846935466	10.6986279636	12.7174024160
H	11.1798498121	14.1535410825	12.7709426493
H	0.5196099925	2.2824725966	12.8439033372
H	16.9603490320	0.3091120269	12.8010246225
H	13.1075816497	9.7470747940	12.7854434542
O	10.3926018504	13.5661787568	12.8793479667
H	8.9721180282	7.3820760044	12.9774486675
O	16.4073898841	1.1321928771	12.9690296466
O	5.2362380357	6.6809760184	12.8948364803
H	7.2510541724	10.5704369752	12.9998423110
O	12.1976614231	6.5212102657	12.9853959063
H	15.9019047195	4.5189957615	13.0821046021
H	16.7785956339	6.5512645552	13.0472872862
H	2.0615754632	2.3254708926	13.0737692350
O	12.4134070819	10.4302256180	12.9742795232
O	3.9714855588	8.9822898223	13.0729273729
H	4.8024943805	7.5929802836	13.0174364006
H	5.6368071946	2.8330757879	13.1573402975
H	11.3518616404	7.0324435749	13.1389764169
H	17.4951057778	-1.6581383783	13.1757010313
H	17.3783769815	7.9687744159	13.3867651092
H	3.7225610339	0.4361299726	13.4223922113

O	4.9129543418	13.9028947783	13.4248417969
H	13.3516100174	14.9723926918	13.4426166294
H	17.4052575404	4.2805582551	13.4517469738
H	5.1282687526	12.0356908226	13.4445803813
O	15.9847996862	10.4756777812	13.3748454544
H	4.8098619603	10.4539432919	13.4763784792
O	9.7374561319	7.5697027553	13.5940076229
H	3.1232041111	8.8142354140	13.5864093082
O	16.6460157559	4.8919568486	13.6372317519
H	9.9001727011	13.9327440552	13.6561126206
O	5.4874284288	11.1468225959	13.7091540600
O	3.1983379528	1.2210741487	13.7716941164
H	9.7455566837	8.5330141401	13.8459093035
H	16.2822522288	11.3313170750	13.7333336173
O	10.0608294004	2.3930305287	13.8666703220
O	12.7316250254	3.0760919926	13.9978692963
H	11.0392832451	2.5797702746	13.9581307313
H	3.9146856198	1.8702411167	14.0402778093
O	5.3312742579	2.7883423493	14.1166190741
H	5.4682626379	14.2732712565	14.1631871224
H	11.0670863636	10.2611816650	14.1998240834
H	15.1935827034	0.9728314032	14.2543075622
H	13.2972542112	2.3967592676	14.4425694901
H	5.7679927204	5.8549136371	14.3464918460
O	1.6877566934	8.4891818382	14.4304266819
H	9.5862427636	3.0969431616	14.3876896377
H	5.5175098134	3.6804000803	14.5273549686
H	12.4221355970	5.8319150828	14.6041850107
H	12.5900306874	3.8117732076	14.6575328266
H	9.4422158542	0.9809541740	14.6519636564
O	14.3494284790	0.8694355312	14.7764154475
H	6.0936895236	1.4708255403	14.8583314404
O	10.4430293175	9.8725978959	14.8610080704
H	13.1684016229	11.1916424443	14.8647604102
H	15.6538326431	9.2671142436	14.9007101996
O	8.9756139480	0.1351603976	14.9167934078
H	1.4776621364	9.3054341362	14.9585909910
O	0.2918619388	12.7542414135	14.9366698391
H	2.5806216936	15.0576529062	14.9873056577
O	6.1224128726	5.1987479423	15.0102050755
H	9.6453270700	6.9197221113	15.1247660283
H	7.3020833961	0.4636103082	15.1462100779
H	1.8208590452	7.8139032692	15.1658411329
H	7.7543778102	4.5110545824	15.1016757826

H	1.0119992057	13.4233153770	15.1855339423
H	5.7437081154	11.2813551251	15.2860226248
O	6.3331307851	0.6271106424	15.3430281766
H	16.0504349242	4.6818650498	15.3704095965
O	12.2495849820	5.3035374268	15.4338878439
H	14.5514633576	0.1739118582	15.4584094772
H	0.5874927795	11.9061103547	15.3895941872
H	11.0294642764	9.3366701460	15.4595119800
O	8.6126628448	4.1537749192	15.4637930202
H	11.3506938206	5.6193489454	15.7082115940
H	9.2755168562	10.9700360371	15.7271170162
O	13.6118457359	11.2854356251	15.7368506920
O	2.1577884840	14.4943396562	15.7112339162
H	14.3006568839	10.5580436090	15.7481279290
H	15.9503531251	13.2489501080	15.8390721764
H	9.2142266882	-0.0797645218	15.8519468631
O	15.5172406206	9.3654333835	15.8716734908
H	9.3062274774	5.6119684565	15.9379727411
H	6.0149285641	5.5636264102	15.9335052228
O	9.7471399291	6.5131962806	16.0417585952
H	2.9019876862	14.0140807463	16.1763558010
H	14.4925294893	12.7939999904	16.1965599547
O	0.9122848730	10.4577492089	16.1829923288
O	5.9592023504	11.4543571347	16.2675713413
H	6.9467024354	11.5898771339	16.2773822633
O	15.6672272714	4.6180855189	16.2808148524
H	15.2263390496	8.4916235789	16.2673511741
H	0.0210318040	10.0342903711	16.3332690483
O	8.7312448214	11.4159341102	16.4325172798
O	15.1718048644	13.4853605663	16.4258294212
H	8.3491302106	3.8163796935	16.3770383450
H	15.2223947716	5.4822284576	16.4537352642
H	9.0603193294	12.3454918894	16.5146158236
O	11.9581489678	8.2215180428	16.5735219537
H	18.6020590649	0.9999675724	16.6468082283
O	1.9288840874	7.0647085914	16.6725685374
H	13.5238267806	7.3089218855	16.7689661993
H	1.8413325457	6.0819949218	16.8175282474
H	11.2313605107	7.6160117859	16.8318215068
H	4.6952875905	12.5271419926	16.8717681066
H	6.1315261463	1.2958743028	16.9104568927
O	14.4722756868	7.0998929682	16.9926660168
H	12.8278993348	4.5937524357	17.0457746574
H	1.3351227549	10.6302510056	17.0635417415

H	2.7864070336	7.2983089965	17.1379309503
H	1.6214158176	3.5823940670	17.2089292490
H	8.8322313037	7.3416924079	17.2525573849
H	15.6919564375	3.2715835292	17.2501439996
O	3.9539031013	13.0859092004	17.2479168459
O	18.3699798380	1.7621839847	17.2546020988
H	12.8772754368	10.8043565970	17.2807484121
H	5.9630060334	10.1771121351	17.2791172128
H	12.1194315983	8.8899704779	17.3076219467
H	17.3809484288	1.7965427895	17.3501985993
O	9.3419390159	13.9286869597	17.4857454313
O	1.8628961867	4.4727440456	17.5728270213
H	15.0199871612	1.8871127214	17.6176367616
H	3.2864051579	12.4706284930	17.6551018465
O	6.1998372575	5.8797802261	17.6620277388
H	10.3013960043	13.7465723591	17.7611720288
O	6.0384335289	1.7389502913	17.8082974288
O	15.8000732947	2.4372671107	17.8103802395
H	0.6971640306	7.9006325469	17.8130505894
H	6.8767107206	8.9872515887	17.8504020210
H	6.9013554561	6.5664628712	17.8349219895
O	11.9494775989	-1.1597934150	17.9181130903
O	8.2098209434	7.7458333678	17.9298775519
H	-1.1078368495	12.8379854652	17.9319643363
O	12.7355475042	4.2128364681	17.9521997186
H	14.4656691887	6.9058969338	17.9610686285
H	9.4910312766	10.9300431858	17.9782265910
O	6.0274803653	9.4731157309	18.0072186185
H	2.7412276806	4.2871208384	18.0059346354
H	12.3278580691	-0.2567330084	18.0237361109
H	4.7446482211	8.3394303632	18.0605903566
O	7.8480253082	3.6588206907	17.9922990518
H	5.3676866645	6.2811689113	18.0288797174
H	7.2732626008	2.8355586219	18.0494393274
O	12.6385835965	10.2880839711	18.1003576681
H	7.1930131540	4.4040463989	18.0423695401
H	8.7433691723	13.6027631769	18.1990865852
O	4.0938643165	7.5944024474	18.2244943666
H	12.6639917770	2.5135155133	18.2656124026
H	4.8090360891	3.0492299883	18.3394724714
H	5.7071827118	1.0167249469	18.4184613894
H	11.0450059341	4.6285763818	18.4788687615
O	1.9522395300	11.5969045247	18.4715683561
H	13.4069304877	4.6941970680	18.5230735584

H	9.4596745226	1.0937314417	18.5634416940
H	13.5056802047	10.1472105213	18.5669436469
H	4.5672414568	13.9869195748	18.5804277631
H	9.4662955376	4.3415592343	18.5927299060
H	10.9324659443	10.7698673325	18.6189915408
H	1.0493369605	12.0086945874	18.6149310081
O	12.5223710095	1.5941404819	18.6326288843
O	0.2016383049	8.1211448861	18.6476449510
O	-0.6599227798	12.4220219638	18.7236215979
H	2.1452345815	1.4415023164	18.7241750440
O	10.1691441062	5.0210772613	18.7295057661
H	8.7159952619	7.8535241278	18.7614815713
H	12.1429451064	-1.6226798207	18.7736310559
H	17.6467046988	5.0582240623	18.7944641307
O	9.9740007072	10.8554467271	18.8458076628
O	4.1375041902	3.5417382197	18.8802466347
H	11.5533416536	1.5895635470	18.8943652103
H	15.8654094732	9.3220922538	18.9387460810
H	0.0471636064	7.1992181702	19.0171576021
H	3.5808515988	7.8864682476	19.0305505730
H	15.6197317730	10.8744553587	19.1449647577
H	3.5785984077	2.8032040749	19.2716950439
O	15.1991939451	9.9765299721	19.2829999931
O	4.9277056477	14.5545234929	19.3253573569
O	9.9014355522	1.4405971187	19.3664292943
H	2.3156364933	11.3720125836	19.3613122899
H	-0.6814051446	13.1233520810	19.4457881943
H	16.5414835803	2.2825256213	19.5165872151
O	14.3585961991	5.7064049314	19.5244541196
O	17.1599249162	5.5305797314	19.5229389974
H	6.0544752358	10.3093063159	19.5270402430
O	2.5796250773	1.4507274373	19.6319289665
H	4.1333329201	15.0235911422	19.6765669412
H	15.3102251077	5.4832048246	19.6902425772
O	7.4654059648	13.1853066281	19.7008329708
H	1.6150530855	8.5098513878	19.7156208145
H	6.6827533336	13.7849383267	19.7327468222
H	9.6344738757	9.6865134277	19.8526694209
H	7.0688469132	12.2996565027	19.9286809295
H	9.9048284221	0.6356034923	19.9760503066
H	10.1921590520	5.8314185630	20.1128599880
H	13.2494641160	1.6289543910	20.1553535680
H	1.8009681330	1.6895705480	20.1926957540
H	17.8461048070	5.5931460250	20.2455330030

O	2.4525235930	8.4914578510	20.2590349670
H	4.6224561770	4.4389398640	20.3099296480
O	16.9629528460	1.9992610800	20.3669775500
O	6.0219902860	10.8514376700	20.3703844900
H	13.8755799260	5.6721997870	20.3976485300
O	12.1430606630	12.6202913170	20.5305367360
O	9.5909909100	8.9336488210	20.5431821110
H	8.9378267900	-1.1557767570	20.5847863110
H	-0.4488855670	0.3722653040	20.6074088370
H	11.2437678690	13.0420781000	20.6689282410
H	10.1057199500	7.3195891040	20.7385730010
H	9.1413779630	2.5626482320	20.7568710470
O	-0.5879251980	-0.6140023070	20.7747326660
H	2.3742706080	7.6272869040	20.7774577080
H	4.2618373230	10.9728502320	20.7917199240
H	12.0421938910	11.6853359340	20.8524192080
O	9.8214076810	-0.7796516400	20.8626265770
H	2.9971951280	10.0105980870	20.8662328850
H	14.8055840230	9.4238069920	20.8963871000
O	10.3062959090	6.3713692780	20.9665819590
O	3.2954429550	10.9520058150	21.0569482950
H	6.4648956700	10.2888008000	21.0611830290
H	16.5463182400	2.5852804890	21.0648352440
O	13.5533183060	1.7140479020	21.1117578150
H	8.8005656560	9.0865245770	21.1359501830
O	4.6186585330	4.8723859980	21.2111755330
H	3.0631029730	5.5723521670	21.2582767920
H	0.2255336520	-0.9092251130	21.2755237040
O	2.1766346640	6.0455078440	21.2845324020
H	13.3773791540	13.3819234780	21.3725507560
H	11.0867870740	9.5860785470	21.3761118680
H	13.7144334280	0.7890759170	21.4521117290
H	11.8281519000	5.8943136120	21.4580191380
H	14.9295658610	13.8709005780	21.5051671240
O	9.1259191430	2.8307352030	21.7052284600
H	2.1842951860	12.6730365450	21.7116083920
H	14.7248589750	3.0210646590	21.7345138460
O	12.6783208070	5.4549878350	21.7634050560
O	14.4939384480	9.0599380010	21.7781420890
O	11.7943481830	10.1541657240	21.7874748620
H	14.8030875920	8.1265726320	21.7908214630
H	12.6229272530	9.6165172930	21.8433583460
H	5.8239836770	6.0382422200	21.8465593020
H	4.6048809590	4.1019668740	21.8573443100

H	9.7905662830	-0.6453798860	21.8585439040
H	12.3516964830	2.4190234850	21.9215572200
O	14.0315672740	13.8864815610	21.9502317700
H	10.0921412030	2.8137245510	21.9855966910
O	15.5085791250	3.4640188780	22.1513805910
O	1.6245265810	13.3436881730	22.1603804270
O	7.3805200000	9.1738012520	22.1813762870
H	9.3320921650	5.7749454420	22.2196890260
H	6.9792956750	8.2597446160	22.2219151470
H	1.8711572500	6.0240095590	22.2413258900
H	15.7165042050	5.4090015380	22.3243188510
H	12.0176536860	3.8799656370	22.3797678140
H	8.7110648650	4.3709739580	22.4082539480
O	6.4276831650	6.5905835560	22.4196157380
O	11.7007003490	2.9392443370	22.4977244660
H	7.2628478890	6.0334269430	22.5499488370
H	12.9250178690	5.9374398180	22.5992182230
O	15.8153861120	6.3544987940	22.6033499130
H	7.7971727590	2.3061049320	22.7687561040
H	2.5046186810	0.1549993760	22.8158500030
O	8.7086598260	5.2760561610	22.8316779500
H	3.3569267020	10.8401849350	22.9183680380
H	1.2497775520	12.8694667880	22.9642891590
O	4.5649115600	2.8318309450	23.0266053590
H	15.5655170670	3.0148448340	23.0406349550
H	5.5362675480	2.5256671150	23.0986726050
H	3.4452893790	1.4651689610	23.1150955060
H	7.6205211320	9.4093402950	23.1187429750
H	15.2896299760	9.8010477420	23.1845189710
H	10.9859782850	10.9857450820	23.1943719440
H	15.0603273440	6.4753133490	23.2542607490
H	14.1554723260	-1.7383002370	23.3899455450
O	7.0743691220	2.2002321350	23.4376916500
H	17.2811008460	6.3908920900	23.4407128500
O	2.8632592790	0.7537486070	23.5150379210
O	9.8749631060	-0.7045829980	23.5223176940
H	10.2508512110	12.3421446540	23.6673093990
O	18.2214202780	6.2003598220	23.7507242550
H	8.9241199620	-0.5788531810	23.8163103550
O	3.1051701020	10.6291014400	23.8564875080
H	11.6861451960	1.9035987610	23.8820118270
H	4.4360874930	3.3197415500	23.8839386020
H	7.2474935820	1.3313621850	23.8861985150
H	5.6974264880	6.6796050310	23.8973427930

O	10.4560017310	11.3995190630	23.9279420550
H	10.4149490150	-0.0131589680	23.9952225780
O	15.6580940770	10.2411986640	24.0029522420
H	18.6693597610	7.0368035420	24.0597345300
H	0.0041577470	11.2908392750	24.0745019270
O	13.6123522150	6.4899646140	24.0834991610
H	3.0451679190	8.9688940110	24.2170886800
H	1.5722666320	11.3682328780	24.2576225040
H	14.7292585250	-2.9670312190	24.2724661020
O	0.7413483700	11.9195753520	24.3211119490
O	14.2294369520	-2.1118969410	24.3235657880
H	4.0121551310	14.5861041090	24.4233622130
H	6.4659900650	14.2065156930	24.4422772800
O	7.4236455710	14.4593684660	24.4891279090
H	9.1109444520	10.3920370700	24.4927624680
H	3.7302493720	11.0688061010	24.5034799910
H	9.1149479800	5.1390940390	24.5904797970
H	13.7270760400	5.6475102820	24.6209505290
H	3.7775498910	7.5913670380	24.6451109410
O	15.5781841070	2.2720517980	24.6519981740
H	15.8431276240	9.5186898970	24.6543771830
O	2.8961492480	8.0827721560	24.6545342600
O	11.5645453420	1.2779656090	24.6569806430
H	1.6829053230	1.5257340020	24.6624133920
H	13.3934376300	7.1947259080	24.7505597050
O	5.1609267540	6.6855326160	24.7550131890
O	8.3665171330	9.8010768960	24.7936881150
H	15.0400535900	1.4743905590	24.9284807890
H	16.5205310120	2.0906353240	24.9505142630
H	1.4691427160	5.1029675600	24.9677355220
O	4.7191093790	14.2393869040	25.0425683800
H	14.1807162940	-0.5711229270	25.1577687650
H	4.7335638590	5.1034909170	25.1644975100
H	13.0673425590	0.5776683470	25.1995577030
H	14.6022181010	3.5554213030	25.2052572990
H	11.4295444000	11.8511340510	25.2557148460
H	12.8772119510	12.4265580080	25.3190565550
H	11.3732107840	1.9278328140	25.3872626410
O	1.1217967210	1.9008188530	25.3932240020
H	7.6912759230	14.2552759800	25.4443088860
H	7.8604510820	10.3498584090	25.4573687490
H	1.3575988940	2.8781561900	25.4653410420
O	4.3911824350	4.2126764040	25.4885212340
H	5.7360027610	7.0408267450	25.4959220040

O	9.3620161250	5.1175183030	25.5567033340
O	14.0672563290	4.3199267460	25.5669674120
O	13.9527893210	0.2970726870	25.5842669720
H	3.3909484280	4.3508775450	25.6052513010
H	4.8512444540	15.0161650760	25.6639739580
O	1.7789762950	4.4801377560	25.6946832010
H	4.4504997650	12.7567341950	25.7171071460
H	13.1566166700	3.9858221880	25.8298006120
H	9.3832096960	6.0711691910	25.8461513600
H	8.9868905940	8.4784273420	25.8688744550
H	10.8313840610	4.1746826290	25.8857258320
O	12.0833023640	12.2481111690	25.9050368690
O	4.4096409160	11.7748656710	25.9605976320
O	16.5245301010	8.4416996520	26.0502683430
H	17.3038072080	12.3640327790	26.0746464070
H	4.9268020060	2.5230117350	26.1286495920
H	2.4829982860	8.5717690180	26.1519189590
O	11.5346252000	3.5538810660	26.2296446100
O	13.3545051940	7.9958918050	26.3015612660
H	17.4614863600	8.5165800810	26.3966566620
H	16.1656586030	7.5887062010	26.4020509730
H	6.1019113480	-3.3322673540	26.5058768200
O	9.1224502810	7.7285843080	26.5137981220
H	15.0233219450	5.4346656670	26.5464198680
H	1.4861295120	4.9002906110	26.5725871960
O	4.9427606870	1.6729103570	26.6301403350
H	3.7867390130	11.6972875490	26.7186883490
O	7.0468894000	-3.4702584830	26.7864306800
H	13.1542705580	8.7978213700	26.8237442630
H	13.9904591860	7.4584109770	26.8257265210
H	8.5023452190	4.1847426840	26.8446994010
H	7.4199362670	-2.5477546200	26.8795525260
H	1.9292526200	1.4371977740	26.9002534780
O	8.0958575420	-0.9281473720	26.9557956620
H	7.4568741420	7.4411877700	26.9625262690
H	11.3330168660	-1.2958719290	26.9809174200
O	2.0016487470	8.8379878900	27.0024721820
O	6.4638909240	7.3350731490	27.0420645530
O	17.1345522930	12.4310678150	27.0522163680
H	15.8675621540	9.5952807450	27.0640186960
H	12.5176385920	11.1820095210	27.0727424730
H	4.0325005090	1.6240949280	27.0805084770
H	9.0819631780	-0.8985646160	27.1318600570
H	5.1216591490	4.5890459440	27.1644990030

H	2.1844348710	9.7949233500	27.1852578660
H	9.7822368990	8.0499159220	27.1904023770
O	15.3554400580	6.1162501390	27.1936436700
H	11.4782943110	3.6542830500	27.2255139220
H	16.4737361700	11.7039091050	27.2692812220
H	13.8816489280	-0.0786992680	27.3110914220
H	15.6316789670	2.8834529020	27.3212241590
H	6.2794885870	6.4073025130	27.3755568030
H	7.7379563220	-0.1194540300	27.4422656910
H	14.6063198900	10.4175474510	27.5867822590
H	6.3213046290	1.4779441600	27.6242211070
O	10.6899545870	-0.8969308280	27.6263004820
O	2.5546156740	1.3476514940	27.6755683230
H	7.9502044960	2.8948024590	27.7007460710
O	8.0896348340	3.8879759230	27.7029838030
O	15.5849407760	10.3039037260	27.7205765170
H	1.4429087040	11.8003647460	27.7240285860
O	12.8289660310	10.5264079080	27.7783517370
H	6.5371377090	4.4450661640	27.8634631590
H	0.0571908330	5.6299133120	27.8763159980
O	5.6494933300	4.9181184600	27.9378713640
H	14.8410218940	5.8326719970	28.0202497750
O	7.2320981760	1.3514909240	28.0205359160
O	1.0284333150	5.5285150270	28.0279092230
O	2.3258530280	11.4299419020	28.0599386770
H	11.0879701360	-0.0987435470	28.0699875870
H	-0.1842257710	-0.7346543220	28.0842266640
O	16.2248652620	2.8424592110	28.0900521470
H	16.4546491290	1.8905216990	28.1665444340
H	11.4255310890	9.4296041250	28.2497475870
O	13.9184929100	-0.2283247590	28.2989271910
H	2.2966038980	2.0812575810	28.3303778530
H	3.0449818860	-0.1420189840	28.3524736580
H	2.5407033070	8.1143974770	28.4255412550
H	7.0034010670	10.7980942140	28.5220496680
O	10.8923824780	8.6406861050	28.5277390050
H	1.1459042720	4.6760256670	28.5359451510
H	5.8721794300	7.9307809600	28.5596040680
H	13.0598670370	11.0849996430	28.5775620290
H	2.9659091620	-1.7324495530	28.5790826920
H	-1.4913688180	-0.0569632190	28.6228562120
H	13.2256380280	0.3923258630	28.6857522420
O	-0.4891634690	-0.0132757290	28.6874617050
O	3.4294481360	-0.9057189470	28.8736747730

O	11.6071937910	3.7924298430	28.9198351660
H	10.2792281840	12.7945681810	28.9553896630
H	2.1374630320	11.0191871980	28.9573923610
H	7.1255324970	1.2262096710	29.0160559980
H	12.4596805960	4.3125574550	29.0662641630
H	11.7314000550	2.0909827510	29.0675812240
O	13.8432934100	5.2340504260	29.1751024410
O	11.7977650740	1.0982328000	29.1819589430
H	8.7373428070	4.4877124420	29.2150007060
H	13.7210379520	13.0423778820	29.2254356940
H	10.2486422900	8.9743792160	29.2275674090
H	15.4317714910	9.2903264830	29.2352815430
O	1.9878187810	3.3332782550	29.3148338990
O	2.7861565020	7.7899578030	29.3458419660
H	2.3348418400	6.9211199490	29.3800935200
H	10.8850263920	4.2593152640	29.4104151780
H	4.7860421080	4.7290015320	29.4923813640
O	5.5984820660	8.1647357630	29.4931390190
O	6.9093627550	10.6022849770	29.4957213750
H	4.6250923160	7.9725830660	29.5276370320
H	6.3417795700	9.7842667130	29.5345178040
H	12.1243826110	7.8919972800	29.5376586100
H	13.4622122490	6.0834727620	29.5589276140
H	2.8140731060	3.7662140040	29.6826564950
O	9.9932403170	12.2459752640	29.7343634110
O	13.7561217060	12.1996430190	29.7599860130
H	4.8328105720	12.9999258160	29.8011991080
H	14.4917218600	4.8722632430	29.8600455940
O	15.2671400630	8.7234676380	30.0364735660
O	9.1833057220	4.7418071500	30.0698049240
H	1.4256709100	3.0718785560	30.1050823210
O	12.8037916970	7.4844041630	30.1532772710
H	5.8596593520	11.8438767530	30.1564291450
H	11.6006287610	0.9704621060	30.1597949340
H	13.6102548020	8.0746600200	30.1629797780
H	8.5248549490	9.9524430530	30.1869976640
H	17.0579253610	14.7209281250	30.2678381270
O	4.2069681900	4.5461881970	30.2823195900
H	2.1908364780	9.2677601400	30.2845178990
H	10.7314337120	12.3153231670	30.3738821300
H	8.7161828160	5.5533535060	30.3928438360
H	3.1858522250	14.4252242960	30.4445202180
H	13.0549611830	12.2538996630	30.4713876520
O	1.8403041040	10.1752745680	30.4901175930

O	9.2018928840	9.2923984050	30.4993420460
H	6.8402678210	7.3364062290	30.5280292270
O	5.2113284220	12.4929889290	30.5512518270
H	8.6897256700	12.9477867000	30.6202637840
O	7.1537313270	15.7570441120	30.6603596470
H	8.6671964270	8.4568858260	30.6637026740
H	4.5891029190	3.7438034430	30.7392919470
H	-0.4255342120	7.3876171590	30.7434279020
H	15.5855591540	9.2814929590	30.7943302300
H	7.3509945910	14.8065093200	30.9264735520
O	7.6983341080	7.0836018110	30.9665617980
H	15.0818214930	12.5012719720	31.0023842180
H	6.2850071170	16.0581791600	31.0586731640
H	16.1935035720	3.7876051310	31.0681388740
O	15.5261643620	4.5345065260	31.0860442210
H	2.4607616180	10.6021686500	31.1638581870
H	9.0516483900	3.4379939080	31.2157788920
O	17.3149756970	14.7251177110	31.2495857180
H	18.3209829820	14.7032869640	31.2520259320
H	16.0618570710	5.3783234070	31.2571632930
H	4.1224836540	1.5560006380	31.2666918820
O	2.9568126010	14.9560993220	31.2674652280
H	8.3132312200	2.0154132470	31.3208573400
O	8.1010829980	13.3141667730	31.3475586570
H	3.1617359110	5.6069478730	31.3802154330
O	0.4221560470	2.7196285150	31.4016756900
H	0.2772616510	1.7362908850	31.4365518030
H	0.7832004290	6.6107863410	31.4644539240
H	17.4225284430	10.0767824450	31.4696741980
O	-0.1908671510	6.7854820560	31.4838711500
O	4.8731875470	2.1769816110	31.4844523860
H	12.2134444210	6.4647731710	31.5018873570
H	4.1222647480	11.8465952810	31.5786151490
H	16.3116489630	13.3129878680	31.6229392390
O	9.0208262800	2.5898987610	31.7476158130
H	10.3976196470	1.7200810710	31.7606434380
O	11.1467545270	1.0401536620	31.7964272010
H	10.2994192390	9.6584353290	31.8155016920
O	15.6430726300	12.5982364780	31.8212901880
O	12.0331864050	12.1885846830	31.8714930730
H	7.5691618310	7.2185051800	31.9475213290
O	2.4469853930	5.9424996460	31.9805472300
H	16.2918067190	11.0445476570	32.0147178050
O	16.6055983880	10.0903841740	32.0518518360

H	3.3399908480	14.4870641210	32.0560630990
H	8.7648023970	13.5272316440	32.0664561310
H	7.3529719000	11.9567629480	32.1270128590
H	11.7336097320	11.2680419450	32.1726551460
O	3.4029279100	11.5249256770	32.2232062180
H	0.8826690460	3.0060542070	32.2511478600
O	11.9130648650	5.9512416390	32.2971199190
H	14.5143562490	4.2607205240	32.2982872680
H	11.8321837590	1.3911406170	32.4375862530
H	4.7656644650	2.2874125850	32.4714907630
H	12.6167572320	5.2674931620	32.5182558190
H	2.2287742170	5.1530713690	32.5606490830
H	12.6468817010	12.5664723980	32.5817915380
O	10.8433933420	9.9713748820	32.5947973490
H	10.7331576290	13.0576631830	32.7015061900
H	10.4865416030	14.5758702380	32.7064859120
H	3.8719211870	10.8888062630	32.8360557740
O	6.9889752330	11.3577388630	32.8437917300
H	10.3737317430	5.5823831150	32.9158054100
H	11.3434956070	9.1852754090	33.0057412510
H	2.8286902740	7.2610937960	33.0463646360
O	13.8398689890	4.2537975480	33.0569931270
O	10.1281927630	13.7271784340	33.1212999610
H	15.4928447010	8.6827732930	33.1965286370
H	14.5066641850	-1.6759947570	33.1970271530
H	5.7428338690	10.2216685510	33.2059949250
H	13.6295593570	3.3062859530	33.2706303430
H	3.4485760840	-1.8174097550	33.2755664530
H	7.7584919900	10.8933057910	33.2924728470
O	9.5158275980	5.4431768950	33.4069247750
H	0.2020233280	0.0838060230	33.4127363990
H	11.9833804530	7.2255139300	33.4285257330
H	3.7785133830	8.4975872450	33.4524521820
O	1.7689092590	3.7405468240	33.4619659460
H	8.8288246990	2.6175530070	33.4650564140
H	6.8719492360	5.0232639320	33.5369018320
H	0.4239779520	9.8560730440	33.6220152830
O	4.9512084650	9.7933064850	33.6325956750
O	13.6715297530	-1.4508019780	33.6831120800
H	8.5974527710	6.7958430520	33.6841372960
O	7.8249206620	7.4411362240	33.7011950640
O	3.7035941600	-0.9464762570	33.7048010360
O	2.9689420100	7.9878339100	33.7194891000
O	12.8916373990	1.7653772260	33.7199608540

H	2.5344996780	3.1544603070	33.7266384440
H	9.6264498890	10.0682231090	33.7842005090
O	14.8715123880	8.3050926710	33.8517622590
H	8.2004719230	8.3502991290	33.8784216920
H	6.5201052810	-2.2215311660	33.8847538540
H	4.6881591810	-1.0784862430	33.9076495150
H	13.0208119730	8.2658930980	33.9145759340
O	12.0499340070	8.0786619850	33.9480853630
O	3.9050237950	2.1005737100	33.9958356980
H	13.2702855430	0.9076755450	34.0032392110
H	3.6310015000	1.1614276410	34.0452463440
H	15.1784317750	7.3703799550	34.0510411600
O	6.5098685230	4.3303728900	34.1171277780
H	1.7214350150	9.1621190450	34.1746734570
H	9.7468452290	4.9237409110	34.1970351030
H	14.6694957590	5.1689903480	34.1980064480
O	8.7517229430	9.9597815810	34.2608527860
H	1.2801723730	3.9976777600	34.3078107120
H	7.2905655770	3.7396702250	34.3094232400
O	0.1148886160	-0.1095169090	34.3668889200
O	0.9808490290	9.7763776110	34.4401033590
O	8.7161623020	2.6702394360	34.4584316770
H	12.2926358170	2.0962707750	34.4732436970
H	13.6401925940	-2.0285149750	34.4880151020
O	6.1909046260	-1.4978390800	34.5118592020
H	5.1659755920	9.7183729880	34.6068842480
H	8.3839772540	1.7729893080	34.7604233790
O	15.1967710540	5.8085112820	34.7654686870
H	10.3464463660	13.6070827610	34.8411335090
H	0.7888968050	0.4450581050	34.8460581680
H	4.1992474980	2.3308486260	34.9351015250
H	7.3892168280	-0.3712090880	34.9963055570
H	0.4796839410	12.8980429520	34.9993108010
H	16.0440497720	5.3203788390	35.0161960600
H	8.7985013410	10.4318466110	35.1478743090
H	0.8556819120	11.3486933660	35.1970737900
H	10.3203695200	2.6693774740	35.2289767140
H	3.4055533290	7.3914538130	35.3182954820
H	5.9435284360	-1.9838612970	35.3600292980
H	7.3433358050	7.2758929630	35.3781653660
H	15.4709287280	0.3358090900	35.3833268560
H	14.5764145470	8.9976759320	35.4103946520
H	3.2307998520	13.5778517430	35.4589572310
O	7.9509715830	0.2874263610	35.5057540690

H	5.7956443090	4.8967067460	35.5386688650
O	11.2531006940	2.6724208470	35.5841390850
O	0.3453328440	4.5175504240	35.5931069240
O	0.6699981040	12.2039857590	35.6778653500
H	8.7869349140	-0.2051168460	35.7433924120
H	10.5111366200	7.7230215070	35.8048344240
O	10.2689269020	13.5602935840	35.8370009830
O	13.2715080060	11.6968531590	36.0053226030
H	17.3593970970	9.1149278830	36.0349911110
H	11.3910551670	3.5701487110	36.0445837800
H	14.4262003370	6.2061738910	36.1508220360
H	13.4718122070	10.7216692740	36.1569171230
H	0.0449872250	3.7692556150	36.1710794670
H	11.0347469080	14.1011415120	36.2140812320
H	0.7314858000	5.2431776390	36.2228490690
O	18.8855117190	15.9198561210	36.2469767460
O	15.0183955360	0.4459134140	36.2513023500
O	3.6578278270	7.2586332440	36.2752254610
H	4.2815938910	6.4715322450	36.2853043090
H	2.1731142190	12.8428621330	36.3143381570
H	12.3670331690	11.8624629050	36.3292809120
O	5.3903287040	9.3863443570	36.3358306670
O	14.2937977450	9.2445688660	36.3367414880
O	7.2996973990	7.3589276830	36.3746141700
H	9.3328589400	12.1893265030	36.4011449270
O	2.9941062190	13.4070050080	36.4114661800
O	5.4088171650	5.1868669310	36.4211254340
H	6.1961397440	8.8086816580	36.4388636860
H	4.6562591030	8.7159835460	36.4648023890
H	9.1605067210	7.3508503470	36.5402714280
H	11.8253761930	1.2585489020	36.5470131040
O	8.7828209230	11.3752832470	36.5688157100
H	13.1297559850	0.3823839710	36.5725213960
O	10.1446892820	7.2633907090	36.5844688280
H	19.2036734250	15.0457724890	36.5947265320
O	4.2895130430	2.5816261210	36.5975112050
H	6.7765196950	6.5748168080	36.6649439140
H	13.9187719550	8.3925207800	36.6799687170
H	3.3720528310	2.2266799280	36.7041094670
H	15.2350808810	-0.4465698580	36.7093333310
H	6.7559914790	0.5252087240	36.7288291660
H	10.9339883170	5.7300431870	36.7330576790
H	4.9741361700	4.3605964120	36.7457731280
H	16.2617040560	12.4467786340	36.7731867930

H	5.3560706690	11.1261769170	36.7840766360
H	18.1297165220	16.2393403400	36.8094854560
H	4.4763832190	12.4440130960	36.8280742020
O	5.4170580570	12.1180962080	36.8290709140
O	12.1776185930	0.3753854840	36.8315033940
O	11.5657749800	4.9647668900	36.8330842050
H	7.9120580900	11.7051024760	36.8572933550
H	12.4444129450	5.4162350450	36.8954123510
H	-1.1180751700	1.6542759040	36.8993130630
H	16.0907315760	8.9960352940	36.9184006640
H	4.8941649970	1.8423678320	36.9188167740
H	14.6930578940	12.3632428360	36.9240272080
H	2.1259864320	6.6737395440	36.9243950210
O	17.0584798200	8.8093766780	36.9260186650
O	13.8995607850	6.5203627740	36.9584849320
H	0.6690947440	7.2240395020	37.1062992760
O	1.2046091540	6.3789672520	37.1635383470
O	-0.4214060520	2.3353007220	37.1878331400
O	15.4926124740	12.8407995650	37.2585518520
O	5.9577647790	0.6215557170	37.3214901070
H	5.6346713050	-0.2862660490	37.4599675710
H	14.4702608350	6.3415651470	37.7268598890
H	-0.6277318450	2.5608615450	38.1136821980

2. Computational input for AIMD (CP2K):

```
&GLOBAL
  PROJECT test
  RUN_TYPE MD
  PRINT_LEVEL low
&END GLOBAL
&MOTION
  &MD
    ENSEMBLE NVT
    STEPS    12000
    TIMESTEP 1.0
    TEMPERATURE 300.15
     &THERMOSTAT
       TYPE    NOSE
       REGION    GLOBAL
       &NOSE
         LENGTH                    3
         TIMECON          2.2237606346543473E+01
       &END NOSE
     &END THERMOSTAT
     &PRINT
       &ENERGY    SILENT
       &END ENERGY
     &END PRINT
  &END MD
  &PRINT
     &TRAJECTORY    SILENT
       &EACH
         MD                    5
       &END EACH
     &END TRAJECTORY
     &VELOCITIES    SILENT
       &EACH
         MD                    5
       &END EACH
     &END VELOCITIES
     &RESTART    SILENT
       &EACH
         MD                    10
       &END EACH
     &END RESTART
  &END PRINT
&END MOTION
```

```
&FORCE_EVAL
  METHOD Quickstep
  &DFT
     UKS    T
     CHARGE 0
     POTENTIAL_FILE_NAME ~/cjw/cp2k_exFiles/GTH_POTENTIALS
     BASIS_SET_FILE_NAME ~/cjw/cp2k_exFiles/BASIS_MOLOPT
     WFN_RESTART_FILE_NAME ./phenol-RESTART.wfn
    &MGRID
      CUTOFF 400
      NGRIDS 20
      REL_CUTOFF 40
    &END MGRID
    &QS
      EPS_DEFAULT 1.0E-12
      EPS_PGF_ORB 1.0E-6
      EXTRAPOLATION ASPC
      EXTRAPOLATION_ORDER 3 # find the best for your system
    &END QS
    &SCF
      EPS_SCF 1.0E-5
      MAX_SCF 30
      SCF_GUESS RESTART
       &OT
         PRECONDITIONER FULL_ALL
         MINIMIZER    BROYDEN
         BROYDEN_BETA         0.9
         BROYDEN_SIGMA         0.1
         LINESEARCH    2PNT
       &END OT
       &OUTER_SCF
         EPS_SCF 1.0E-5
         MAX_SCF 5
       &END OUTER_SCF
       &MIXING ON
         ALPHA 0.1
         BETA    0.5
       &END MIXING
       &PRINT
          &RESTART_HISTORY
            FILENAME = MD_RESTART_HISTORY.wfn
            ADD_LAST NUMERIC
            BACKUP_COPIES 5
            &EACH
```

```
            &END EACH
          &END RESTART_HISTORY
      &END PRINT
    &END SCF
    &XC
      &XC_FUNCTIONAL PBE
      &END XC_FUNCTIONAL
      &XC_GRID
        XC_SMOOTH_RHO NN10
        XC_DERIV SPLINE2_SMOOTH
      &END XC_GRID
      &vdW_POTENTIAL
         POTENTIAL_TYPE PAIR_POTENTIAL
         &PAIR_POTENTIAL
            TYPE DFTD3
            PARAMETER_FILE_NAME ~/cjw/cp2k_exFiles/dftd3.dat
            REFERENCE_FUNCTIONAL PBE
            R_CUTOFF 10.
         &END PAIR_POTENTIAL
      &END vdW_POTENTIAL
    &END XC
    &PRINT
      &MULLIKEN    SILENT
        FILENAME =CHARGE.mulliken
        &EACH
          MD                    5
        &END EACH
      &END MULLIKEN
    &END PRINT
  &END DFT
  &SUBSYS
    &CELL
      ABC [angstrom] 17.0434      14.7600      40.0000
      PERIODIC XYZ
    &END CELL
    &TOPOLOGY
      COORD_FILE_NAME init.xyz
      COORD_FILE_FORMAT xyz
    &END TOPOLOGY
    &KIND Fe
      BASIS_SET DZVP-MOLOPT-SR-GTH
      POTENTIAL GTH-PBE-q16
    &END KIND
    &KIND O
```

```
      BASIS_SET DZVP-MOLOPT-SR-GTH
      POTENTIAL GTH-PBE-q6
    &END KIND
    &KIND C
      BASIS_SET DZVP-MOLOPT-SR-GTH
      POTENTIAL GTH-PBE-q4
    &END KIND
    &KIND H
      BASIS_SET DZVP-MOLOPT-SR-GTH
      POTENTIAL GTH-PBE-q1
      MASS 2.0
    &END KIND
    &KIND N
      BASIS_SET DZVP-MOLOPT-SR-GTH
      POTENTIAL GTH-PBE-q5
    &END KIND
  &END SUBSYS
&END FORCE_EVAL
```
